# Synergetic electronic spin modulation and asymmetric orbital hybridization at the CoSe_2_/Fe_3_Se_4_ interface inducing a robust SEI for enhanced sodium ion storage

**DOI:** 10.1039/d6sc03276a

**Published:** 2026-05-25

**Authors:** Mingyu Lian, Yitong Sun, Di Zhou, Yijia Zhang, Qingqing Zhou, Ying Jiang, Zhengqing Ye

**Affiliations:** a Tianjin Key Laboratory of Materials Laminating Fabrication and Interface Control Technology, School of Material Science and Engineering, Hebei University of Technology Tianjin 300401 P.R. China zqye@hebut.edu.cn; b School of Material Science and Engineering, Tianjin University of Technology Tianjin 300384 P.R. China yingjiang@email.tjut.edu.cn; c Joint Key Laboratory of the Ministry of Education, Institute of Applied Physics and Materials Engineering, University of Macau Avenida da Universidade Taipa Macau SAR 999078 P.R. China

## Abstract

Transition metal selenides (TMSes) are recognized as promising anode materials for sodium-ion batteries (SIBs) owing to their ideal capacity and low cost, but their practical application suffers from crucial issues of inferior cycling stability and sluggish reaction kinetics. Herein, we design a magnetic CoSe_2_/Fe_3_Se_4_ (CFSE) heterostructure to simultaneously regulate orbital and spin features, aiming to systematically reveal their synergistic effect on sodium-ion storage. The orbital spin splitting of the CFSE heterostructure drives the Co^2+^ spin state transition from low to high, which improves the adsorption energy and lowers the diffusion energy barrier of sodium ions. Meanwhile, Co–Se–Fe asymmetric orbital hybridization promotes a charge transfer pathway at the interface and ensures the directional migration of ions, thereby inhibiting irreversible structural variations and tailoring an ultrathin and robust SEI film during battery operation. As a result, the as-prepared CFSE electrodes achieve a high reversible capacity of 395.8 mAh g^−1^ at 2.0 A g^−1^ over 1200 cycles and deliver an excellent rate capability of 364.4 mAh g^−1^ at 10.0 A g^−1^. This work provides an in-depth understanding of the spin–orbit modulation mechanism and inspiration for developing advanced conversion-type anodes.

## Abstract 2

Magnetic CoSe_2_/Fe_3_Se_4_ heterostructure with high-spin polarization and Co–Se–Fe asymmetric orbital hybridization lifts Co/Se band centers, thus improving adsorption, reducing diffusion barrier, and promoting the formation of a robust SEI film.

## Graphical abstract

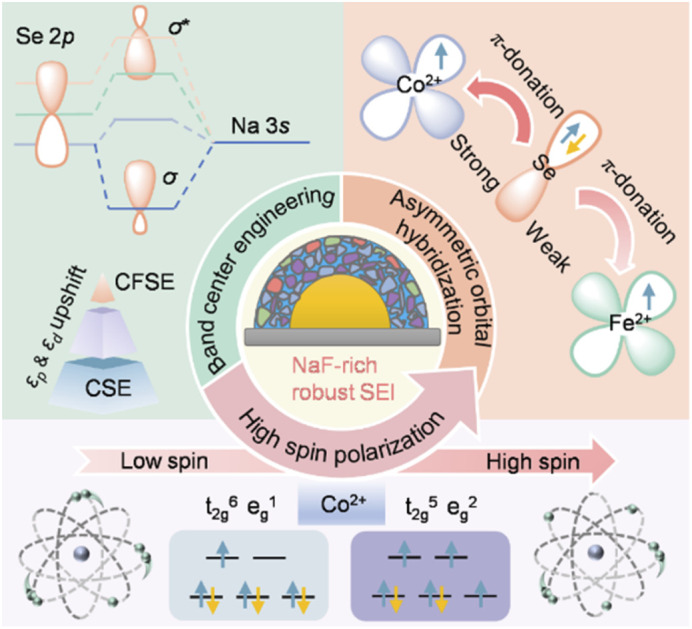

## Introduction

In the pursuit of sustainable energy storage systems, sodium-ion batteries (SIBs) have emerged as a promising alternative to lithium-ion batteries (LIBs) owing to their abundant resources, cost-effectiveness, and environmental compatibility.^1–3^ However, SIB anodes face severe challenges, including large ionic radius of Na^+^ (1.02 Å) compared to Li^+^ (0.76 Å), which results in sluggish diffusion kinetics, thus leading to poor rate capability of SIBs.^4–9^ Therefore, great efforts have been devoted to developing high-rate anode materials for extending the potential application of SIBs. Among them, transition metal selenides (TMSes) have garnered substantial attention owing to their high theoretical capacity and outstanding rate performance originating from their multi-step electronic conversion reactions and narrow bandgap structures.^10–13^ Nevertheless, the development of TMSes has been impeded by the low intrinsic electronic conductivity and slow ion diffusion kinetics. In addition, they also suffer from unfavorable volume variations during discharge/charge cycling, which lead to the repeated breakage/re-formation of the solid electrolyte interphase (SEI) film, thus deteriorating the cycling life.

In this respect, heterostructure engineering can spontaneously generate a built-in electric field (BIEF) at the interface between two phases, which exhibits favorable charge transfer and ion diffusion dynamics.^14–20^ Among them, bimetallic selenide heterostructures including Sb_2_Se_3_/WSe_2_,^21^ Se–CoS_2_/CoSe_2_,^22^ and MoSe_2_/Bi_2_Se_3_ (ref. 23) have gained significant attention owing to their ability to promote fast and durable sodium storage during the discharging and charging processes. For instance, Ge *et al.* developed a microcrystalline-MoSe_2_/amorphous-MoSe_*x*_O_*y*_ (C-MoSe_2_/A-MoSe_*x*_O_*y*_) heterostructure, which shows an ultrahigh specific capacity and rate performance for SIBs.^24^ The improved performance is attributed to the charge self-modulation effect at the microcrystalline-amorphous heterogeneous interface in C-MoSe_2_/A-MoSe_*x*_O_*y*_. This activates Mo–Se bonds and modulates the interfacial charge rearrangement, thus significantly enhancing sodium ion adsorption and electron/ion transport. Li *et al.* successfully designed a CoSe/MoSe_2_ heterostructure integrated with homogeneous carbon composites (CoSe/MoSe_2_-C) through solvothermal methods, followed by selenization and carbonization processes.^25^ As expected, CoSe/MoSe_2_-C shows a high capacity and ultra-long lifespan in sodium-ion half/full batteries. The construction of the heterostructure can regulate the charge redistribution at the heterointerface by virtue of the BIEF effect, which reduces the energy barrier for ion diffusion and enhances charge transfer kinetics. However, considering the spin-sensitive characteristics of sodium storage reactions, there is a critical need to develop new types of heterostructures that can modulate spin states in TMSes.

Spin represents an additional intrinsic property of electrons and often plays an essential role in the electronic configuration of the active site, further optimizing the sodium ion adsorption energy during battery operation.^26^ For example, Li *et al.* proposed electronic-spin regulation in a CoSe_2_/MoSe_2_ heterostructure toward fast and stable sodium storage.^27^ The introduced Mo cations within the CoSe_2_/MoSe_2_ not only serve as electron donors to modulate charge-spin configurations with rich active electronic states but also lead to the upshift of d/p band centers and the reduction of the Δd–p band center gap, thus improving sodium ion adsorption capacity and decreasing the ion diffusion barrier. Sikandar Iqbal *et al.* introduced a competitive coordination approach to fabricate a Se-rich ZnSe/MnSe heterostructure, enabling high reversibility for sodium ion storage.^28^ In particular, the p orbitals of Se induce a shift from the high-spin to low-spin state of Mn d-orbitals, which accelerates charge migration from ZnSe to MnSe. This provides rich sites for sodium ion insertion/de-insertion and accommodates volume expansion, which in turn improves the reversibility of anode materials during cycling. Consequently, constructing unique TMSe heterostructures to concurrently modulate both electron density and spin state is expected to be an effective way to improve electrochemical performance of SIBs. Although spin engineering helps understand the origins of favorable sodium storage performance in heterostructures, the spin state modulation mechanism remains unclear. Moreover, the interplay of spin and orbitals at the heterointerface can tailor the atomic coordination environment and electronic structure compared to their bulk counterparts, resulting in unique physicochemical characteristics.

Herein, we prepared a magnetic CoSe_2_/Fe_3_Se_4_ (CFSE) heterostructure with synergistic regulation of spin state and orbital hybridization, intending to systematically reveal their synergistic influence on sodium ion storage. Theoretical calculation demonstrates that the orbital spin splitting of the CFSE heterostructure induces a spin-state transition in Co^2+^ (from low-spin to high-spin), which results in an upshift in the d-band center (*ε*_d_) of Co, thus optimizing the adsorption energy and stabilizing the Na-intercalated phase. Meanwhile, asymmetric Co–Se–Fe orbital hybridization at the heterointerface leads to the generation of an electronic transport pathway, promoting charge transport from Fe to Co and lowering the diffusion barrier for Na^+^, thereby improving the electrochemical reaction kinetics of CFSE. Moreover, the CFSE heterostructure reduces the dissociation energy barrier of NaPF_6_, inducing an even and inorganic-rich component SEI film to improve structural stability. Ultimately, the CFSE electrodes achieve fantastic electrochemical performances for sodium storage compared to those of pristine individual components.

## Results and discussion

**Fig. 1:**
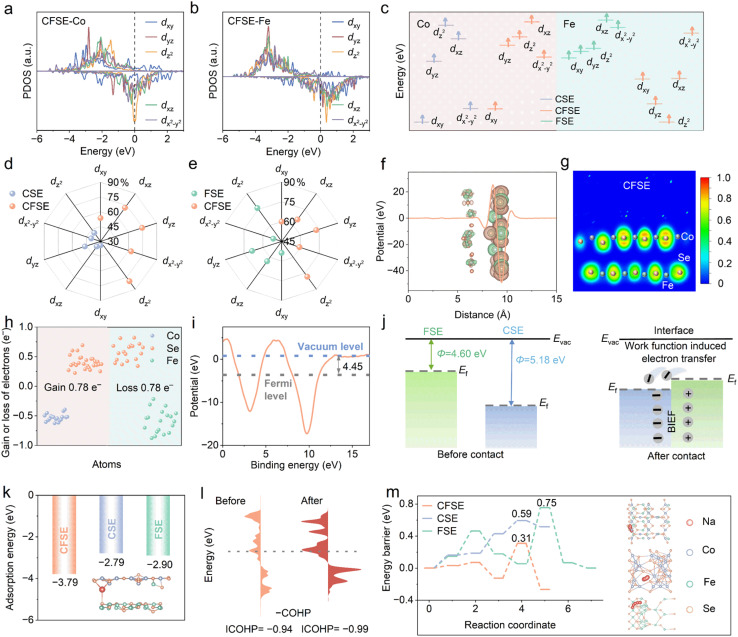
(a) The PDOS of Co-d orbitals and (b) Fe-d orbitals in CFSE. (c) Schematic diagram of the energy levels of Co-d and Fe-d orbitals. (d and e) Spin polarizability of splitting 3d orbitals of various samples. (f) Charge density difference of the heterointerface in CFSE and corresponding planar average charge density difference in the *Z* direction. (g) The Electron Localization Function (ELF) of CFSE. (h) Electron gain/loss of different atoms calculated by Bader charge analysis. (i) The work function of CFSE. (j) Schematic diagram of electron redistribution at the heterointerface between FSE and CSE. (k) Na^+^ adsorption energy. (l) COHP plots and corresponding ICOHP values of CFSE before and after Na insertion. (m) The top views of diffusion pathways and diffusion barriers of Na^+^ in CFSE, CSE, and FSE.

To study the effect of the heterostructure on the electronic structures, the total density of states (TDOS) and partial density of states (PDOS) of the three samples are calculated. The DOS results show that CFSE possesses larger density states at the Fermi level (*E*_f_) compared with CoSe_2_ (CSE) and Fe_3_Se_4_ (FSE), suggesting better electronic conductivity, which is beneficial for charge transport during redox reaction processes (Fig. S1). In addition, the PDOS results reveal that the electronic states of Fe-d orbitals in FSE and Co-d orbitals in CSE show asymmetry characteristics, which originate from the uneven arrangement of electrons in spin-up and spin-down states (Fig. S2). In comparison, this asymmetry of Fe-d orbitals and Co-d orbitals in CFSE is stronger (Fig. 1a and b), thus leading to more spontaneous spin polarization. This result indicates that CFSE can offer spin-polarized electrons, which are conducive to adsorbing sodium ions. Furthermore, there are changes in the electron-donor or electron-acceptor characteristics of the coordination environment in CFSE by virtue of the introduction of spin state manipulation as well as an improvement in Co d-band centers (Fig. S3).

Moreover, the energy levels of 3d_*yz*_, 3d_*xz*_, 3d_*xy*_, 3d_*x*^2^–*y*^2^_, and 3d_*z*^2^_ are plotted according to their respective d-band centers (Fig. 1c and S4). For Co-d spin-up orbitals, the 3d_*z*^2^_ orbital is the highest occupied orbital in CSE, while the 3d_*xz*_ orbital is the highest occupied orbital in CFSE. Notably, the construction of heterostructures can significantly influence the highest occupied orbitals of active metal atoms owing to the orbital hybridization and charge transport across the heterointerface.^29^ It is considered that the electrons occupying orbitals near the *E*_f_ possess higher reactivity. Therefore, the highest occupied d orbital plays a significant role in modulating the adsorption behavior of intermediates. Furthermore, the PDOS of the three samples were simulated to investigate electron contributions from various Co-3d orbitals and Fe-3d orbitals (Fig. 1d and e). In CSE, 39.8% and 40.7% of electrons in the d_*x*^2^–*y*^2^_ and d_*z*^2^_ orbitals are unpaired, while 34.1%, 36.3%, and 46.6% of electrons in d_*xy*_, d_*xz*_, and d_*yz*_ orbitals are unpaired. Moreover, in CFSE, 62.5% and 79.9% electrons in the d_*x*^2^–*y*^2^_ and d_*z*^2^_ orbitals are unpaired, and 53.7%, 72.2%, and 74.3% electrons are unpaired in the d_*xy*_, d_*xz*_, and d_*yz*_ orbitals, respectively. Thus, the probability of identifying Co^2+^ in the t_2g_^5^e_g_^2^ high electronic spin state is higher in CFSE than in CSE.

To further confirm the charge regulation effect of the active Fe–Se and Co–Se bonds at the heterointerface, differential charge density was calculated. The green regions indicate electron depletion, while the orange regions represent electron accumulation. The heterointerface coupling allows electron redistribution and the generation of a BIEF, which is further substantiated by the quantized electron distribution along the *Z*-axis direction (Fig. 1f). The observed peaks located at the interlayer positions confirm the substantial charge transfer at the heterointerface, indicating obvious charge redistribution. Electron localization function (ELF) results demonstrate reduced electron localization on Se atoms in CFSE compared with pristine FSE (Fig. 1g and S5), which is consistent with predominant charge transfer from Fe_3_Se_4_ to CoSe_2_. The charge redistribution near the CFSE interface and the electron depletion and accumulation among different atoms were quantified *via* Bader charge analysis (Fig. 1h). Bader charge analysis indicates that the CoSe_2_ unit gains 0.78 electrons from the Fe_3_Se_4_ structure.^30^ The generation of a BIEF induces space charge redistribution, which is expected to be an effective approach for manipulating spin states, thus accelerating Na^+^ migration and enhancing the rate performance of SIBs.^24^ To elucidate the intrinsic driving mechanism of electron transfer at the interface, the work function value (*Φ*) of different samples was further obtained by DFT calculations (Fig. 1i and S6). The *Φ* of CFSE (4.45 eV) is the lowest, which indicates that electrons on the heterostructure surface are the easiest to transfer, enabling CFSE to exhibit superior electrical conductivity. In addition, FSE shows a lower *Φ* of 4.60 eV compared to 5.18 eV for CSE, facilitating a spontaneous electron flow from FSE to CSE at the heterointerface, thereby generating a BIEF from FSE to CSE that significantly accelerates reaction kinetics and the reversible insertion/extraction process of Na ions (Fig. 1j).

The adsorption energy values and corresponding structural models of Na^+^ on the surfaces of CFSE, CSE, and FSE are shown in Fig. 1k and S7. Notably, the CFSE heterostructure exhibits a more negative Na adsorption energy (−3.79 eV) than individual CSE (−2.79 eV) and FSE (−2.90 eV), indicating that heterostructure construction can induce stronger adsorption of Na atom and thermodynamically more favorable Na adsorption.^31^ The bonding strength of Na–Se was further investigated through Crystal Orbital Hamilton Population (COHP) analysis. The negative and positive values of COHP signify bonding and antibonding states, respectively. The bonding states of CFSE are mostly below the *E*_f_ with higher occupation, while the bonding states of CSE and FSE shift below the *E*_f_ with lower occupation. Furthermore, the integrated COHP (ICOHP) was calculated to directly quantify the bonding strength. CFSE exhibits a more negative ICOHP value (−0.38 eV) than CSE (−0.30 eV) and FSE (−0.31 eV), confirming its stronger chemical bonding (Fig. S8).

COHP analysis is also employed to assess the bonding stability of the corresponding Na-intercalated phase. The ICOHP value in Na-intercalated CFSE (−0.99 eV) is markedly more negative than that of pristine CFSE (−0.94 eV), indicating substantially enhanced thermodynamic stability after Na intercalation (Fig. 1l). The reaction kinetics are further analyzed. Fig. 1m shows the diffusion paths of sodium ions and the corresponding diffusion energy barriers in the three materials (Fig. S9). The results indicate that CFSE has the lowest diffusion barrier for sodium ions, of only 0.31 eV, suggesting relatively fast migration of sodium ions within CFSE. This improves electrochemical reaction dynamics, thus implying the potential for CFSE to achieve exceptional electrochemical properties.^32^

**Fig. 2:**
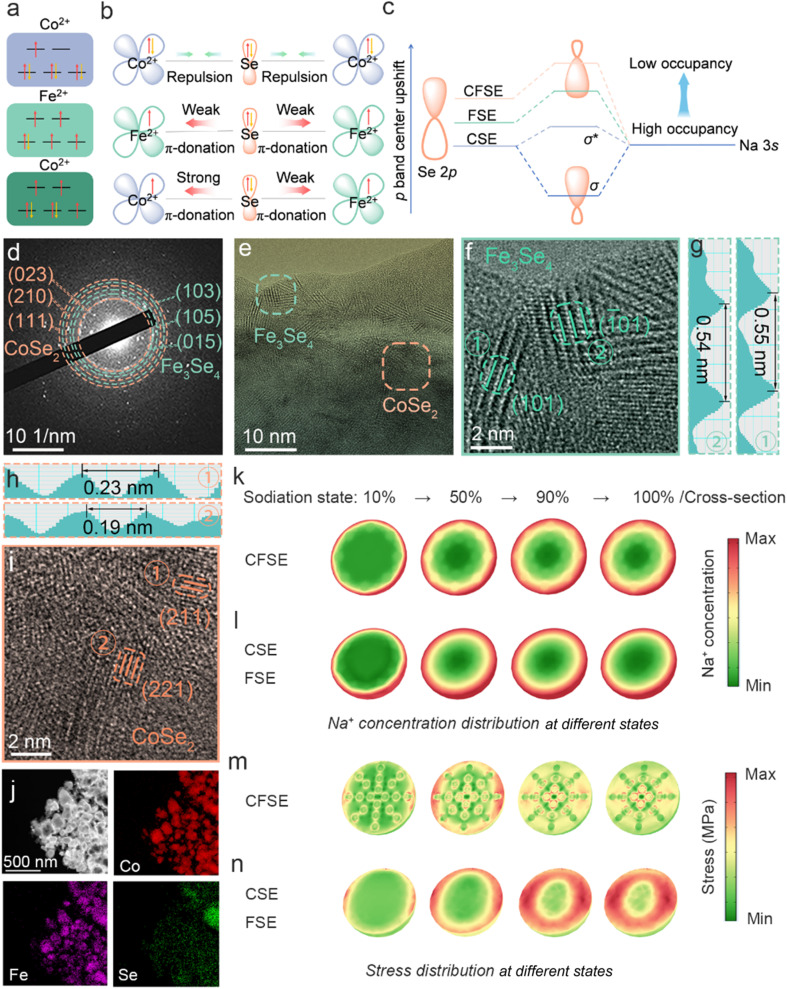
(a) Schematic illustration of the energy-level splitting of d orbitals in different metal ions. (b) Illustration of electronic interaction in the t_2g_ orbitals for CSE, FSE, and CFSE. (c) Schematic illustration of the p band center modulation and Na–Se interaction. (d) SAED pattern of CFSE. (e, f and i) HRTEM images and (g and h) FFT lattice images of the selected areas of CFSE. (j) HAADF-STEM image and corresponding elemental mapping of CFSE. The Na^+^ concentration and stress distribution of the (k and m) CFSE model and (l and n) CSE or FSE model at sodiation states of 10%, 50%, 90%, and 100%, respectively.

Notably, CFSE exhibits the highest Na^+^ binding energy and the smallest Na^+^ diffusion barrier. This is primarily caused by its unique electronic structure (Fig. 2a). PDOS analysis results demonstrate that the electron structure of Co cations in CSE is in the low spin state (t_2g_^6^e_g_^1^). In contrast, Co cations in CFSE possess a valence electron configuration of t_2g_^5^e_g_^2^ with a high spin state (Fig. 2b). More specifically, the valence electron configuration of Co^2+^ in CSE is 3d^7^ with fully occupied π-symmetry (t_2g_) orbitals. Consequently, electron repulsion emerges as the primary interaction between bridging Se and Co. In contrast, the π-symmetry orbitals of Co^2+^ in CFSE are half-empty, allowing interaction with bridging Se *via* π-donation.^33^ The electronic configuration of Fe^2+^ can be described as t_2g_^4^e_g_^2^ in both CFSE and FSE, which is unpaired for the high spin state of Fe^2+^. The interaction between Fe^2+^ and Se is implemented through the π-donation.^34^ In addition, the effect of Co and Fe on Se site activity can be assessed through the covalent character.^35^ The covalent characters of Fe–Se and Co–Se bonds are 87.8% and 89.4%, respectively (Fig. S10). The higher covalent character indicates that the metal 3d orbitals have a stronger electron withdrawing ability, which results in charge transfer between Se sites and metal atoms. CFSE possesses a stronger π-donation interaction of Co–Se than its Fe–Se counterpart, thus leading to partial transfer of Fe electron to Co *via* asymmetric orbital hybridization within the Co–Se–Fe bridging configuration. The Co–Se–Fe asymmetric orbital hybridization in the heterostructure drives electron redistribution and regulates the electron-spin state in the metal center, resulting in the upshift of the *ε*_d_ of Co (Fig. S3). Moreover, Se atoms in CFSE possess the highest p band center (*ε*_p_) among all samples (Fig. S11), thus reducing the occupancy degree of the Na–Se antibonding sigma orbital (σ*) (Fig. 2c). This result improves the interaction between Na^+^ and Se in the metal selenide heterostructure, thereby enhancing adsorption energy toward Na^+^.^36^

Inspired by theoretical calculations, the CFSE anode material was successfully constructed by a simple two-step strategy of co-precipitation and selenization (Fig. S12). The selected-area electron diffraction (SAED) pattern exhibits the presence of the (023), (210), and (111) planes of CoSe_2_ as well as the (103), (105), and (015) crystal planes of Fe_3_Se_4_ (Fig. 2d). This confirms the generation of CFSE heterostructures. In the high-resolution transmission electron microscopy (HRTEM) images of CFSE, as shown in Fig. 2e, the marked lattice spacings of 0.54 and 0.55 nm correspond to the (101) and (1̄01) planes of Fe_3_Se_4_ (Fig. 2f and g). Additionally, CFSE features *d*-spacings of 0.19 and 0.23 nm, corresponding to the (221) and (211) planes of CoSe_2_ (Fig. 2h and i). The presence of the heterojunction was further confirmed by high-angle annular dark-field scanning TEM (HAADF-STEM) and corresponding elemental mapping analysis (Fig. 2j), revealing the uniform distribution of cobalt, iron, and selenium elements in CFSE.

To further investigate the influence of heterostructure design on ion diffusion kinetics and mechanical stress distribution, the finite element analysis was employed. Two different models were constructed: (1) a heterostructure CFSE composite (uniformly dispersed CSE-inserted particles were fabricated on an FSE substrate), and (2) a control model of CSE or FSE with a single particle phase. The sodium ion concentration of both systems at various sodiation states is investigated. The single-phase particles show a larger radial sodium ion concentration gradient compared to the heterostructure during discharging (Fig. 2k and l), indicating that the single-phase particles are prone to localized concentration accumulation. Specifically, surface-preferential Na^+^ insertion during discharging establishes a Na^+^ concentration gradient, inducing substantial mismatch strain at the bulk-surface interface. The single CSE or FSE system, featured by slow surface diffusion dynamics, exhibits incomplete bulk sodiation and gradual stress accumulation (Fig. 2l and n). In contrast, CFSE exhibits highly efficient and even bulk-surface kinetics, significantly alleviating concentration polarization and associated mechanical stress (Fig. 2k and m). Therefore, strategic regulation of surface Na^+^ transport kinetics by compositional heterostructure engineering provides a significant approach for achieving improved diffusion dynamics throughout the whole particle in anode materials.

**Fig. 3:**
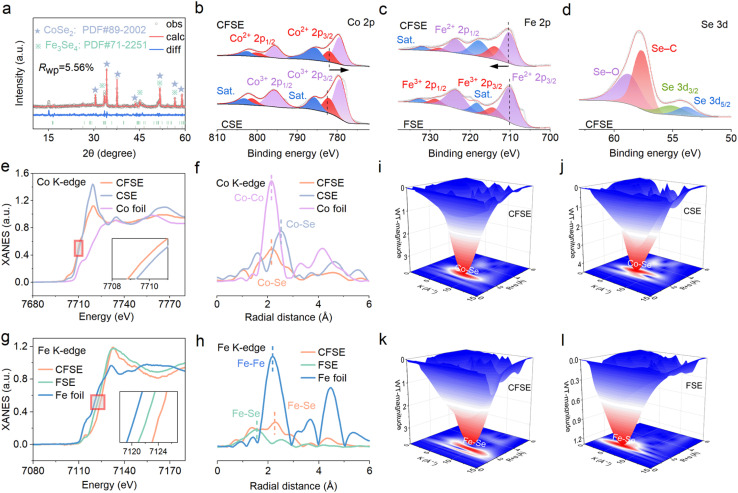
(a) XRD pattern of CFSE. High-resolution XPS spectra of (b) Co 2p, (c) Fe 2p, and (d) Se 3d. (e) Co K-edge XANES spectra. (f) Co K-edge FT-EXAFS spectra. (g) Fe K-edge XANES spectra. (h) Fe K-edge FT-EXAFS spectra. WT contour plots of the Co K-edge in (i) CFSE, (j) CSE, and the Fe K-edge in (k) CFSE and (l) FSE.

X-ray diffraction (XRD) was used to investigate the crystalline phase structure of the as-synthesized CFSE samples. As shown in Fig. 3a, CFSE exhibits representative diffraction peaks at 33.5°, 51.7°, and 56.6°, corresponding to the (202), (310), and (1̄16) planes of the Fe_3_Se_4_ phase (PDF no. 71-2251). Meanwhile, three dominant peaks located at about 34.2°, 37.6°, and 51.7° refer to the (210), (211), and (311) planes of CoSe_2_ (PDF no. 89-2002). The XRD analysis results confirm the successful preparation of CFSE. X-ray photoelectron spectroscopy (XPS) was employed to explore the surface compositions and chemical bonding states in the three samples (Fig. 3b–d). The survey spectra of CFSE samples exhibit the presence of Fe, Co, O, N, C, and Se elements (Fig. S13). Specifically, the Co 2p spectra (Fig. 3b) of CFSE exhibited two peaks at 799.9 and 782.0 eV, which are attributed to Co^2+^ 2p_1/2_ and Co^2+^ 2p_3/2_, respectively. The peaks at 779.5, 795.7, 802.4, and 785.4 eV are ascribed to the 2p_3/2_ and 2p_1/2_ of Co^3+^ and two satellite peaks, respectively.^37^ In comparison to CSE, the Co 2p peak of CFSE exhibits a shift toward lower binding energy, which could be attributed to the formation of a BIEF within the heterostructure, suggesting that the charge transfer results from the rearrangement of electron cloud density due to the robust synergistic coupling between CoSe_2_ and Fe_3_Se_4_.^38,39^ The Fe 2p XPS spectra (Fig. 3c) of CFSE can be divided into six distinct peaks, where the peaks emerging at 713.9 and 727.8 eV are assigned to 2p_3/2_ and 2p_1/2_ of Fe^3+^, while the peaks centered at 710.4 and 723.7 eV belong to Fe^2+^ 2p_3/2_ and Fe^2+^ 2p_1/2_, respectively. Additionally, two remaining peaks located at 717.9 and 732.2 eV correspond to satellite peaks.^40^ The positive shift of the Fe 2p peak results from the valence structure reconstruction induced by the incorporation of CoSe_2_, which indicates the reduction of valence electrons compared to the *E*_f_ and downshift of the d band center. In the high-resolution Se 3d spectra (Fig. 3d and S14), two distinct peaks located at 53.8 and 55.1 eV are attributed to the Se 3d_5/2_ and Se 3d_3/2_, respectively. The peak at 57.6 eV is associated with the Se–C bonding configuration. The peak observed at 58.8 eV corresponds to SeO_*x*_ species, resulting from the oxidation of Se^2−^ under air conditions.^41^

To further identify the generation of the Co–Se–Fe bond, the detailed structures of CSE, CFSF, and FSE were analyzed by X-ray absorption spectroscopy (XAS) characterization. Compared with CSE (Fig. 3e), the X-ray absorption near-edge structure (XANES) of the Co K-edge in CFSE moves to a lower energy, demonstrating the increased electron density on Co atoms due to the heterostructure effect, which corresponds well with the XPS and DFT results. Nevertheless, the near-edge characteristics of the Fe K-edge in CFSE are obviously different from those of its Co K-edge counterpart, implying an elevated oxidation state of Fe species (Fig. 3g). In addition, the weakened peak intensity of the white line near 7720 eV (Fig. 3e) indicates that the white line peak intensity is associated with the spin state of metal atoms and the coordination environment.^42^ Specifically, Co^2+^ exhibits a higher spin state in CFSE than in CSE.

The Fourier transformed extended X-ray absorption fine structure (FT-EXAFS) spectra of CFSE show a dominant peak at 2.21 Å, corresponding to the first shell Co–Se coordination. The Co–Se coordination in CFSE exhibits a low shift of the characteristic peak compared to CSE, which can be attributed to the reduced bond length. This result indicates enhanced coordination interaction between Co and Se in CFSE, which is consistent with DFT calculation results. The lower peak intensities could originate from interfacial lattice disordering of CFSE. An obvious weakening in the intensity of the Co–Se peak for CFSE compared with CSE (Fig. 3f) suggests a lower coordination number that is likely related to the variation of spin state in CFSE (Fig. S15 and Table S1). However, the peak intensity of Fe–Se coordination located at about 2.20 Å in CFSE is higher than that in FSE, indicating a higher Fe–Se coordination number after introducing heterostructure engineering (Fig. 3h). The high radical distance shift of the Fe–Se peak in CFSE could be attributed to the generation of strongly coupled interfaces between CoSe_2_ and Fe_3_Se_4_.

Moreover, the *k*^3^-weighted wavelet transform (WT) contour plot is utilized to further validate the local coordinated environment of CSE, CFSE, and FSE, respectively (Fig. 3i–l). In contrast to CSE, the WT contour plots of CFSE exhibit a minor shift to the right direction, indicating that there exists electron transfer between Fe_3_Se_4_ and CoSe_2_ to promote the sodium-ion adsorption/desorption processes (Fig. 3i and j). Similarly, the maximum intensity of FSE centers at *k* = 5.33 Å^−1^, while the highest intensity in CFSE exhibits an obvious positive shift (Fig. 3k and l). This result further demonstrates that the BIEF at the CFSE heterogeneous interface leads to the variation in the Fe electron configuration. Interestingly, the notably greater intensity of Fe–Se in CFSE compared to FSE reveals an augmented coordination number. The above results indicate the successful modulation of the local coordination environment for the Co and Fe sites in CFSE by the BIEF within the heterostructure.

**Fig. 4:**
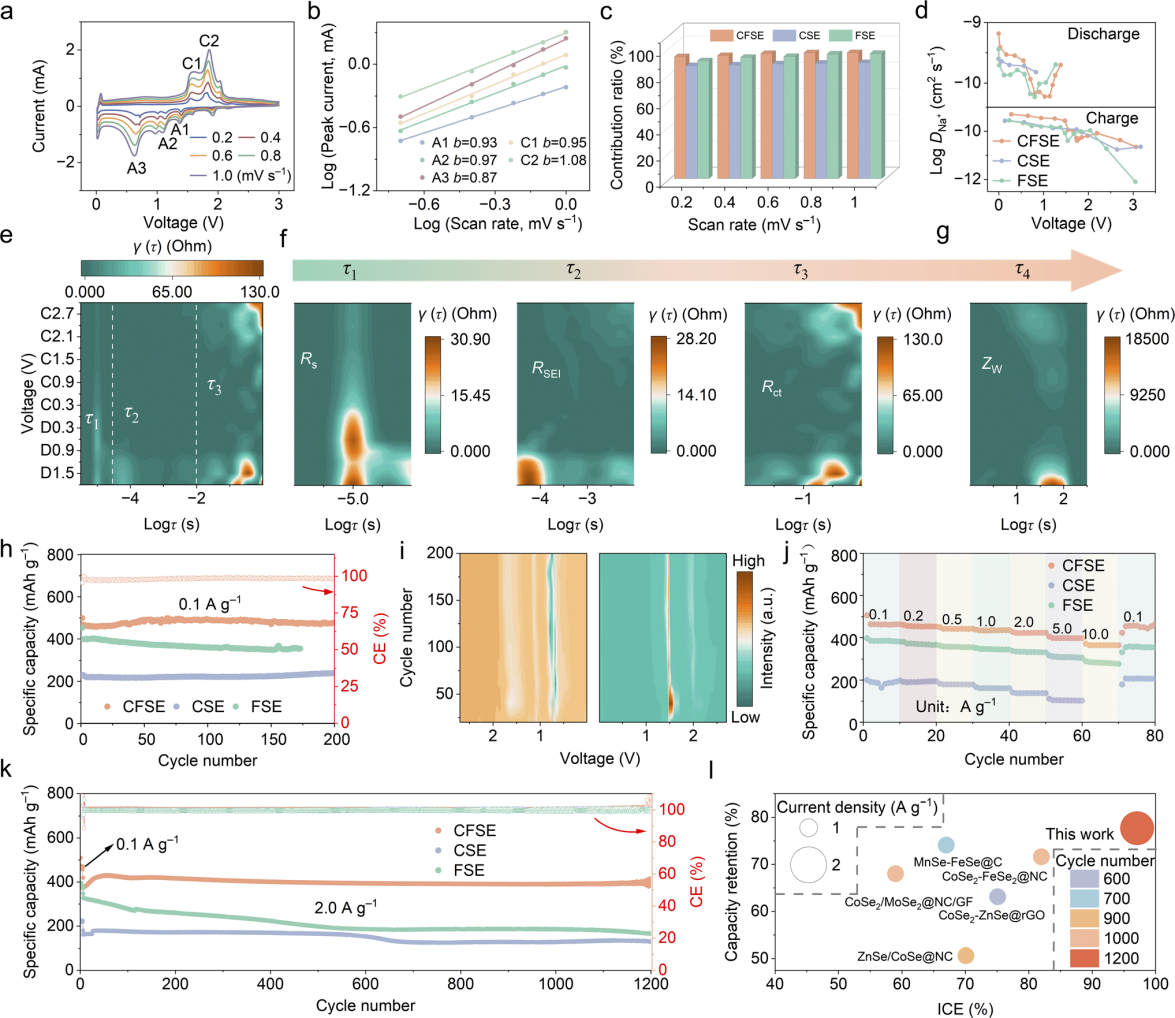
(a) The CV curves of CFSE at different scanning rates. (b) The fitting curves and corresponding *b* values of each marked peak in (a), derived from the logarithmic values of different peak currents and scan rates. (c) Contribution ratios of capacitive behaviors at various scan rates for CFSE, CSE, and FSE. (d) Diffusion coefficients of CFSE, CSE, and FSE during discharge and charge processes. (e–g) 2D DRT intensity mapping originating from *in situ* EIS measurements during the first discharge and charge processes of CFSE. (h) Cycling performance at 0.1 A g^−1^. (i) 2D d*Q*/d*V* contour plot of CFSE during 200 cycles. (j) Rate performance. (k) The long-term cycling performance of CFSE, CSE, and FSE at 2.0 A g^−1^. (l) Comparison of long-term cycling performance of CFSE with other conversion anode materials for SIBs.

To further study the electrochemical reaction process within the three samples, the cyclic voltammetry (CV) curves at different scan rates from 0.2 to 1.0 mV s^−1^ were measured. As illustrated in Fig. 4a, the CV curves of CFSE exhibit excellent similarity in peak positions and shapes within the voltage range of 0.01 to 3.0 V at various scan rates, demonstrating the superior conversion reaction kinetics of the heterostructure during charge and discharge processes. The correlation between the peak currents and different scan rates is calculated.^15^ The electrochemical kinetic behavior during discharge/charge processes can be evaluated by the *b* values. Fig. 4b reveals that the *b* values for the anodic and cathodic peaks of CFSE are 0.93 (A1), 0.97 (A2), 0.87 (A3), 0.95 (C1), and 1.08 (C2), respectively, which are approximately close to 1.0, suggesting the predominance of the pseudocapacitive reaction mechanism for SIBs based on the CFSE electrode.^38^ Furthermore, the *b* values for pristine CSE and FSE are both about 1.0 (Fig. S16 and S17), indicating the dominant pseudocapacitive contribution of the composite structure. Notably, the *b* values of CSE and FSE are obviously smaller than that of CFSE, indicating that although pseudocapacitive behavior is dominant, the diffusion-controlled contributions cannot be ignored (Fig. S18). Additionally, CFSE displays a higher pseudocapacitive contribution (96%) than that of CSE (88%) and FSE (95%) at a scan rate of 1.0 mV s^−1^ (Fig. S19).^43^ Furthermore, CFSE shows higher capacitive contribution ratios than those of CSE and FSE electrodes at all investigated scan rates (Fig. 4c). These results indicate excellent charge storage kinetics of CFSE anodes, which are conducive to their superior rate capability and long-cycle stability.

The ion diffusion coefficient is a crucial factor that directly reflects the dynamics of electrochemical reactions. To further assess the Na^+^ diffusion coefficient (*D*_Na^+^_) for CFSE, CSE, and FSE electrodes, the galvanostatic intermittent titration technique (GITT) was conducted at a current density of 0.1 A g^−1^ (Fig. S20).^44^ As a result, CFSE shows a higher average *D*_Na^+^_ value compared with CSE and FSE during the discharge/charge process (Fig. 4d), suggesting the dominance of rapid Na^+^ intercalation/extraction processes at the electrode–electrolyte interface. In addition, the activation energy (*E*_a_) for all electrodes can be further calculated using the Arrhenius equation.^38,45^ An optimal equivalent circuit is adopted to perform simulations of a series of collected electrochemical impedance spectroscopy (EIS) spectra to evaluate specific resistance (Fig. S21 and 22).^46,47^ The *E*_a_ value of CFSE (10.79 kJ mol^−1^) is smaller than those of CSE (26.39 kJ mol^−1^) and FSE (29.17 kJ mol^−1^), demonstrating that the energy barrier of the Na^+^ desolvation in CFSE is relatively lower compared with CSE and FSE, which promotes the Na^+^ intercalation into CFSE.^40^

*In situ* EIS was employed to elucidate Na^+^ transfer kinetics of CFSE electrodes. The EIS profiles exhibit two overlapping semicircles and a line in the high, medium, and low frequency ranges, which correspond to Na^+^ diffusion in the SEI (*R*_SEI_), charge transfer resistance (*R*_ct_), and Warburg impedance (*Z*_w_), respectively (Fig. S23). The semicircular arc of the CFSE electrode in the high-frequency ranges (Fig. S24) shows an obvious shrinkage during the discharge process and then remains stable during the charge stage, indicating effective charge carrier transfer in CFSE anodes. In contrast, the semicircular arc of CSE exhibits a disordered state during the initial discharge process, illustrating slow charge transport and an unstable interface. It is noticed that the *R*_SEI_ and *R*_ct_ values for the CFSE electrode have been significantly reduced when compared to those for the CSE and FSE electrodes (Fig. S24–26), revealing that the spin-modulated CFSE heterostructure can significantly accelerate reaction kinetics of Na^+^ across the SEI layer and Na^+^ transport from the electrolyte to the electrode.^48^

To accurately distinguish various electrochemical processes, *in situ* EIS were further analyzed utilizing the distribution of relaxation times (DRT) technique (Fig. 4e–g). According to the timescale investigation, the peaks predominantly center at *τ*_1_ = 10^−5^, *τ*_2_ = 10^−4^–10^−2^, *τ*_3_ = 10^−2^–1, and *τ*_4_ = 1–2 s, which correspond to the ohmic resistance (*R*_s_), *R*_SEI_, *R*_ct_, and *Z*_W_, respectively. Across *τ*_1_–*τ*_3_ timescales, CFSE shows smaller peak intensities and excellent cycling stability than CSE and FSE. In particular, the *τ*_2_ peak of CSE and FSE is initially large and decreases after the subsequent discharge stage, which is attributed to uneven and thick SEI formation during the electrochemical process (Fig. S25 and S26). In contrast, the *τ*_2_ peak is considerably smaller and more stable, indicating an even and thin SEI film formation in the CFSE electrode material. Notably, two distinct *R*_ct_ peaks are observed, confirming that the electrochemical reaction of CFSE is a multi-electron reaction (Fig. S24). The *τ*_3_ peaks for CFSE are smaller than those of CSE and FSE, which indicates superior Na^+^ transport kinetics and enhanced electronic conductivity (Fig. S27). During the discharging stage, the intensity of the *τ*_3_ peaks for CFSE progressively decreases, accompanied by a more obvious reduction in relaxation time than those of CSE and FSE, suggesting accelerated interfacial kinetics *via* the spin-driven heterostructure.

To illustrate the impact of heterostructure construction and spin modulation on electrochemical performances, the CV curves of CFSE at 0.1 mV s^−1^ between 0.01 and 3.0 V were investigated (Fig. S28). During the first cathodic scan, a reduction peak at ≈1.35 V is detected, which is owing to the insertion of sodium ions into CFSE (CoSe_2_/Fe_3_Se_4_ + *x*Na^+^ + *x*e^−^ → Na_*x*_CoSe_2_/Fe_3_Se_4_). Subsequently, the cathodic peaks located at ≈1.13 and 0.69 V can be attributed to the conversion reaction of the Na-intercalated phase into metal Co, Fe, and Na_2_Se as well as the formation of the SEI film, respectively. A pair of redox peaks near 0.01 V stands for the typical Na^+^ intercalation/extraction processes in the carbon materials.^49^ During the anodic process, the oxidation peaks appear at 1.53 and 1.76 V, which correspond to the formation of CoSe_2_ and Fe_3_Se_4_. Compared to CSE and FSE, the CV curves of CFSE in the subsequent two cycling processes show relatively overlapped shapes, suggesting excellent electrochemical reversibility and stability in CFSE.^27^ Fig. S29 illustrates the galvanostatic charge/discharge (GCD) profiles of the CFSE electrodes for the initial three cycles. For the first cycle, the specific charge/discharge capacities of the CFSE electrode are 484.3/499.1 mAh g^−1^, with an initial coulombic efficiency (ICE) of 97.1%, which is higher than that of CSE (191.6/200.4 mAh g^−1^, 95.6%) and FSE (437.8/471.8 mAh g^−1^, 92.8%). The results demonstrate the excellent energy conversion efficiency and superior energy utilization rate of CFSE anodes.^40^

The cycle performance of CFSE, CSE, and FSE at a current density of 0.1 A g^−1^ is exhibited in Fig. 4h. The capacity of the CFSE electrode shows a gradual increase after the initial cycles, which is attributed to the gradual activation of active materials during the cycling process. After 200 cycles, the CFSE electrode achieves a higher reversible and stable capacity of 475.6 mAh g^−1^ than that of FSE (354.1 mAh g^−1^, 173 cycles) and CSE (240.3 mAh g^−1^). To get a profound understanding of the phase evolution of CFSE electrodes during repeated discharge and charge processes, differential specific capacity (d*Q*/d*V*) curves within the first 200 cycles at 0.1 A g^−1^ are illustrated in Fig. 4i and S30. During the discharging process of the CFSE electrode, a d*Q*/d*V* peak related to the insertion of sodium ions is detected at approximately 1.6 V. The intensity of this peak slightly weakens within the subsequent cycles, indicating that the non-faradaic behavior gradually becomes predominant.^32^ Conversely, the peak at 0.73 V shows high and stable intensity within a narrow voltage range, indicating a continuous conversion reaction during discharge processes, with faradaic behavior being predominant. During the charging process, the peaks are detected in the contour plot at 1.53 and 1.9 V, which stand for the reverse conversion reaction process and the extraction of the Na^+^ phase, respectively. As the cycle number increases, the peak intensity at 1.9 V gradually strengthens, suggesting clearer charge platforms and a more prominent contribution of faradaic behavior. This observation is likely associated with the structural reorganization of CFSE and gradual stabilization of charge transport during repeated cycling. The interface construction of the heterostructure between CoSe_2_ and Fe_3_Se_4_ can more effectively facilitate activation and phase reorganization of the electrode materials, as well as facilitate fast Na^+^ diffusion kinetics. As shown in Fig. 4j, the CFSE electrode achieves reversible specific capacities of 461.7, 454.2, 442.0, 434.8, 422.3, 398.0, and 364.4 mAh g^−1^ from 0.1 to 10.0 A g^−1^, respectively. When the current density switched back to 0.1 A g^−1^, the specific capacity of CFSE can still be retained at 457.2 mAh g^−1^. The corresponding GCD profiles of CFSE display well-maintained potential plateaus, suggesting that the electrode at various current densities possesses low polarization and faster reaction kinetics. In comparison, the CSE and FSE electrodes show inferior rate performance with poor capacity delivery and low capacity retention from 0.1 to 10.0 A g^−1^ due to increased voltage polarization and kinetic barriers (Fig. S31). In addition, it can be detected from Fig. S32 and Table S2 that the rate performance of the CFSE electrode is remarkably superior to earlier reported selenide heterostructure anode materials for SIBs, attributable to the synergistic effect of orbital coupling and spin modulation in the CFSE heterostructure. Moreover, long-term cycling performance assessment reveals that CFSE maintains a significant specific capacity of 395.8 mAh g^−1^ after 1200 cycles at 2.0 A g^−1^, with a capacity retention of 94.4%, surpassing both CSE (131.3 mAh g^−1^) and FSE (167.3 mAh g^−1^), as well as reported selenium-based heterojunction anodes for SIBs (Fig. 4k, l, S33 and Table S3).

**Fig. 5:**
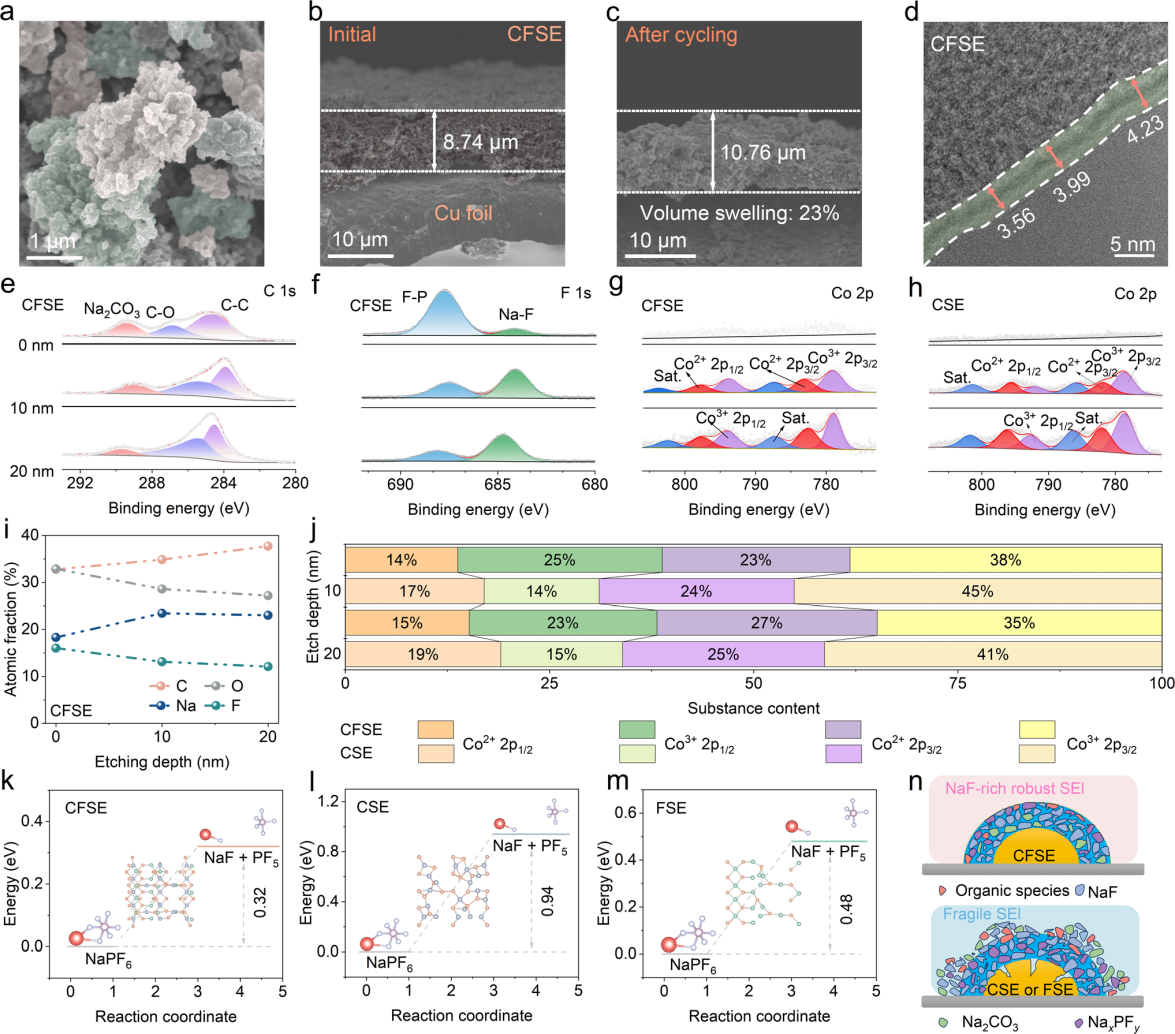
The chemical and mechanical stability of CFSE. (a) SEM image of CFSE after cycling. Cross-sectional SEM images of CFSE (b) before and (c) after cycling. HRTEM images of the (d) CFSE electrode at 0.1 A g^−1^. XPS spectra of (e) C 1s, (f) F 1s, and (g) Co 2p in CFSE electrodes after the 10th cycle. (h) Co 2p XPS spectra of CSE after the 10th cycle. (i) Atomic fractions of C, O, F, and Na at different etching depths. (j) Various Co spin state percentages of CFSE and CSE at different etching depths. The energy illustration of NaPF_6_ decomposition into NaF and PF_5_ on the surfaces of (k) CFSE, (l) CSE, and (m) FSE. (n) Schematic illustration of the SEI structure formed on CFSE and CSE (FSE).

To further understand the excellent cycling stability of the CFSE heterostructure, we conducted morphology characterization of the different anodes collected after 100 cycles at a current density of 1.0 A g^−1^. For CSE and FSE, severe cracks and loosely stacked particles were detected on the entire surface of the electrode (Fig. S35a, b, S36a and b), indicating that the SEI layer generated on the surface could be unstably maintained after continuous charge and discharge cycles. In contrast, the surface of CFSE remained smooth and intact without obvious cracks (Fig. 5a and S34), thus achieving excellent cycle stability for efficient sodium storage. Moreover, the macroscopic changes in CFSE, CSE, and FSE electrodes were investigated by measuring their cross-sectional thicknesses. The initial thickness of the CFSE electrode was 8.7 µm, and after cycling, the thickness increased to 10.8 µm, representing a volume expansion of 23% (Fig. 5b and c). In contrast, the volume variations of CSE (Fig. S35c and d) and FSE (Fig. S36c and d) anodes are much larger than that of the CFSE heterojunction during the repeated charging and discharging processes, which can explain the superior cycling life of the CFSE anodes.

We utilized HRTEM to explore the impact of the heterostructure on the morphological and structural features of the SEI. Specifically, the CFSE electrode retains a thinner and more homogeneous SEI film (∼4 nm) after 10 cycles, compared with the thicker and more uneven SEI films detected on the CSE and FSE electrodes (Fig. 5d and S37). XPS measurements were conducted to further investigate the structure and composition of the SEI on different electrodes after 10 cycles. As depicted in Fig. 5e, the pristine CFSE sample exhibits three primary peaks at approximately 284.6, 286.9, and 289.4 eV, which correspond to C–C, C–O, and Na_2_CO_3_, respectively.^50,51^ After etching, the C 1s spectra peak related to Na_2_CO_3_ in CFSE shows remarkably lower intensity than those of CSE and FSE, indicating less decomposition of electrolyte (Fig. S38). This suggests the formation of stable SEI layers in CFSE.^50^ Furthermore, the O 1s spectra analysis also demonstrates the existence of C–O and C=O bonds, which are attributed to the decomposition of the ether-based electrolyte (Fig. S39). The cycled CFSE anode exhibits a lower peak intensity of oxygen species compared to CSE and FSE, inhibiting continuous electrolyte decomposition.^52^ In addition, the F 1s spectra display the signals of Na–F (684.1 eV) and P–F (687.85 eV), which are attributed to the formation of a NaF-rich SEI and residual sodium salt (Fig. 5f and S40).^53^ As etching continued, the Na–F bond peaks became more prominent, indicating *in situ* generation of a robust NaF-rich SEI inner layer. According to the atomic percentage analysis, the SEI layers of CFSE exhibit lower atomic percentages of O and higher fluorine content, demonstrating a thinner organic outer layer as well as a NaF-rich inorganic inner layer (Fig. 5i and S41). Notably, NaF exhibits a small Na^+^ diffusion barrier and remarkable electronic insulation characteristics, which promote fast Na^+^ migration inside the SEI, while significantly inhibiting electron penetration from the SEI layer.^5^ These properties of the NaF-rich inorganic layer are conducive to the rapid charging capability of the CFSE anode and enable its stability in long-term cycling. Moreover, in the initial unetched state after full sodiation, the signals for Co 2p of CFSE are not observed, which could be attributed to the formation of the NaF-rich SEI layer (Fig. 5g, h and j). As etching proceeded, the intensities of Co^2+^ and Co^3+^ peaks were significantly strengthened and eventually remained stable, indicating an obviously thinner SEI layer. The formation mechanism of the SEI on the surface of CFSE includes NaPF_6_ decomposition into NaF and PF_5_ at the anode–electrolyte interface. DFT calculations demonstrate that the dissociation energy barrier of NaPF_6_ on CFSE is 0.32 eV, significantly lower than that of CSE (0.94 eV) and FSE (0.48 eV) (Fig. 5k–m). This result suggests that CFSE effectively promotes the P–F bond breaking in NaPF_6_ and facilitates NaF nucleation by offering abundant active sites (Co and Se), which is beneficial for the generation of a robust SEI layer on the heterostructure (Fig. S42).

In summary, the Co^2+^ spin state transition from low-spin to high-spin at the heterojunction interface promotes more inorganic (inner: NaF) and less organic (outer layer) dual-layer SEI components. The unique and dense dual-layer SEI structure mitigates the continuous electrolyte consumption and interface side reactions with reduced gas evolution (*i.e.*, the reaction path i–ii) (Fig. S43).^54^ Therefore, spin modulation enables the achievement of a thin and robust dual-layer SEI as well as high ionic conductivity due to the shorter diffusion pathway (Fig. S44), which contributes to achieving stable cycling performances. As illustrated in Fig. 5n, the SEI generated in an ether-based electrolyte exhibits a double-layer architecture containing a dense NaF-rich inorganic inner layer and an organic outer layer. During the Na^+^ insertion/extraction process, the organic outer layer ensures sufficient and stable Na^+^ flux, facilitating rapid sodium storage kinetics. Additionally, the dense inorganic inner layer dominated by NaF/Na_2_CO_3_ has a high modulus to resist severe volume variations and suppress excessive electrolyte decomposition, thereby achieving superior electrochemical performances.

## Conclusion

In this study, we elaborately designed and prepared CFSE heterostructures *via* a facile co-precipitation and selenization strategy. The systematic DFT calculations and experimental studies were adopted to explore the role of electronic-spin regulation and asymmetric orbital hybridization in the enhanced sodium storage performance of the CFSE composite. CFSE shows a high spin state of Co, which contributes to energy level up-shift and activates more electronic states in 3d orbitals. The Co–Se–Fe asymmetric orbital hybridization at the heterointerface accelerates electron transfer from Fe to Co, which results in the upshift in the *ε*_d_ of Co. This enhances the adsorption energy and lowers the diffusion barrier of sodium ions. Furthermore, the synergetic spin-orbital modulation in the CFSE heterostructure reduces the dissociation energy barrier of NaPF_6_, thereby tailoring a stable, thin, and NaF-rich SEI layer, which is conducive to the sustained long cycle life of SIBs. Consequently, compared with CSE and FSE, CFSE achieves a longer cycle life (395.8 mAh g^−1^ after 1200 cycles with a capacity retention of 94.4%). This study highlights the critical roles of spin engineering and orbital hybridization in heterostructure materials, which can offer guidance for developing other conversion-type anodes for high-performance SIBs.

## Author contributions

Mingyu Lian: investigation, formal analysis, data curation, writing – original draft, writing – review & editing. Yitong Sun: investigation, formal analysis, data curation, writing – original draft, writing – review & editing. Di Zhou: data curation, formal analysis, writing – review & editing. Yijia Zhang: data curation, formal analysis, writing – review & editing. Qingqing Zhou: data curation, writing – review & editing. Ying Jiang: software, validation, funding acquisition, project administration, resources, supervision, writing – review & editing. Zhengqing Ye: conceptualization, funding acquisition, investigation, methodology, project administration, resources, supervision, writing – review & editing.

## Conflicts of interest

There are no conflicts of interest to declare.

## Supplementary Material

SC-OLF-D6SC03276A-s001

## Data Availability

The data that support the findings of this study are available from the corresponding author upon reasonable request. Supplementary information (SI) is available. See DOI: https://doi.org/10.1039/d6sc03276a.
