# Supplementary material for: Synergetic electronic spin modulation and asymmetric orbital hybridization at the CoSe2/Fe3Se4 interface inducing a robust SEI for enhanced sodium ion storage
Source: Chem Sci. 2026 May 25;17(27):13606–17. doi: 10.1039/d6sc03276a (PMC13234767; doi:10.1039/d6sc03276a)
Supplement: SC-017-D6SC03276A-s001 [file SC-017-D6SC03276A-s001.pdf]

Supplementary Information (SI) for Chemical Science.
This journal is © The Royal Society of Chemistry 2026

Supporting Information

Synergetic Electronic Spin Modulation and Asymmetric Orbital Hybridization at $CoSe_2/Fe_3Se_4$ Interface Inducing Robust SEI for Enhanced Sodium Ion Storage

Section I. Experimental Section

Chemicals and materials

All chemicals employed in this study are analytical grade and do not require any additional purification procedures.

Synthesis of $CoSe_2/Fe_3Se_4$ (CFSE) composites

0.438 g $Co(NO_3)_2 \cdot 6H_2O$ (Aladdin, 99%) was dissolved in 60 mL deionized water (denoted as Solution A). 15 mg polyvinyl pyrrolidone (PVP, Macklin, GR) and 0.975 g 2-Methylimidazole (Aladdin, 99%) were dissolved in 60 mL deionized water (denoted as Solution B). Solution A was rapidly poured into Solution B. After stirring for 10 min at room temperature, the mixture was allowed to stand for 6 h. Afterwards, the precipitate was washed with deionized water and ethanol, and then dried at 80°C to obtain Co precursor. 0.484 g $K_3[Fe(CN)_6]$ (Macklin) and Co precursor were dissolved in 135 mL deionized water. After stirring at room temperature for 30 min, the mixture was allowed to stand for 12 h. Finally, the precipitate from the previous step was washed with deionized water, and then was dried at the freezer for 36 h to obtain CoFe precursor. To obtain CFSE composites, the Se (Macklin, AR) and as-prepared CoFe precursor were placed in two quartz crucibles, following a mass ratio of 2:1. The powder was calcined at 600°C for 2 h in a tubular furnace, with the heating rate of 2°C min^{-1} under an argon (Ar) atmosphere.

Synthesis of $CoSe_2$ (CSE) composites

The Co precursor was calcined under the above conditions to obtain CSE composites.

Synthesis of Fe_3Se_4 (FSE) composites

0.99 g $K_3[Fe(CN)_6]$ (Macklin) and 1.35 mL hydrochloric acid (12 mol L^{-1}) were dissolved in 135 mL deionized water (denoted as Solution C). Solution C was heated to 60°C and then stirred for 4 h. After cooling to room temperature, it was allowed to stand for 12 h, and washed with deionized water and ethanol, and then dried at 80°C to obtain the Fe precursor. Finally, the powder was calcined under the above conditions to obtain FSE composites.

Electrochemical characterization

Coin cells (CR2032, Canrd) were assembled using CFSE, Na foil, and 1 M $NaPF_6$ in DME, respectively, as the anode, cathode, and electrolyte. Whatman's Glass fiber (GF/D) as the separator. The anode slurry consisted of a 7:2:1 ratio of CFSE, Super P, and carboxymethyl cellulose (CMC). Then the slurry was coated on a copper foil and dried in a vacuum oven at 60°C for 24 h. The production process for FSE and CSE was similar to that of CFSE. The coin cells were evaluated by the NEWARE Battery Test System (CT-4008Tn-5V10mA-164, Shenzhen, China) with a voltage range of 0.01 to 3.0 V to test the cycling and rate performances. The electrochemical impedance spectroscopy (EIS) measurements and the cyclic voltammetry (CV) tests were evaluated by CS2350M (Corrtest) electrochemical workstation. EIS measurements were carried out in the frequency range from 100 kHz to 0.01 Hz. The simulation of EIS spectra was conducted via the Z-view software. The CV analysis was operated between 0.01 and 3.0 V with the various scan rates from 0.1 to 1.0 mV s^{-1}. Galvanostatic intermittence titration technique (GITT) tests were tested for 20 min at

0.1 A g^{-1} and a 60 min relaxation time after the fifth cycles, with a potential range of 0.01-3.0 V.

Physical characterization

X-ray diffraction (XRD) using a Rigaku Smartlab (9 kW) with Cu Kα radiation (λ = 1.5418 Å) at a scan rate of 8° min^{-1} from 10° to 60°. Scanning electron microscopy (SEM) and Transmission electron microscopy (TEM) were performed on JSM-7610F and JEM-2100F instruments to conduct basic morphological characteristics of anode materials. And the high angle annular dark-field (HAADF) STEM was taken under an acceleration voltage of 200 kV. Elemental analysis was performed on an XPS (Cu Kα radiation (λ = 1.5418 Å), Thermo Nexsa G2). The standard processing of XANYS and EXAFS data was performed with the ATHENA program.

First principles calculations

All density-functional theory (DFT) calculations were performed by the Vienna Ab initio Simulation Package (VASP).[1] The electron-ion interaction is simulated using the projector augmented wave (PAW).[2] A plane-wave basis set with a kinetic-energy cutoff of 400 eV was used to expand the wave functions. The electron-electron exchange and correlation interactions are described by the Perdew-Burke-Ernzerh (PBE) functional and Generalized Gradient Approximation (GGA).[3] A 1×1×1 Gamma k-point grid was employed for the Brillouin zone sampling. The energy convergence was fixed to 10^{-5} eV and the force convergence criterion was less than 0.03 eV A^{-1}. During the relaxation for the density of states (DOS) simulations, the van der Waals (vdW)-inclusive DFT-D3 method was considered.[4] The

bonding/antibonding characteristics of the systems were further explored through the Crystal Orbital Hamilton Population (COHP) analysis using the LOBSTER program.

Finite element simulations

This simulation adopts the Solid Mechanics and the physical field of dilute species transport by a finite element analysis based on COMSOL Multiphysics under an adaptive grid. In defining the boundary parameters, the original concentration of Na^+ on the sphere surface was set as 0.5 M. The sodium ion diffusivities are 10^{-11} m^2 s^{-1} in the FSE material and 10^{-10} m^2 s^{-1} in the CSE material. During discharge simulations, the local concentration of inserted Na^+ in the particles is quantified. Subsequently, the strain of particles is coupled to the local concentration distribution, and a stress-strain simulation of the particles is conducted via the Solid Mechanics module in COMSOL.

Section II. Supplementary Figures and Tables

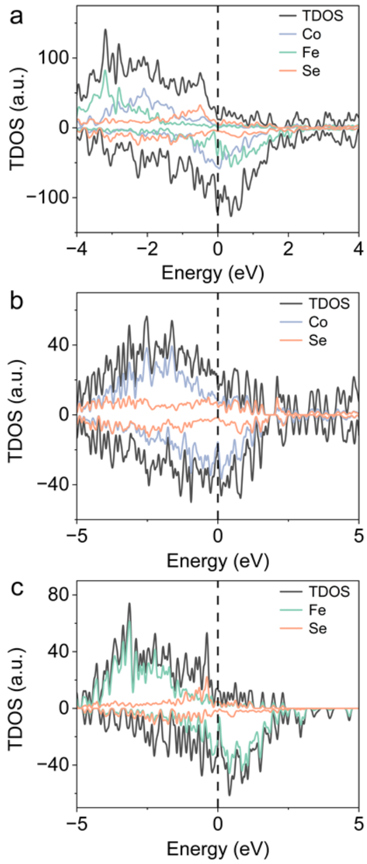

Fig. S1 TDOS plots of (a) CFSE, (b) CSE, and (c) FSE.

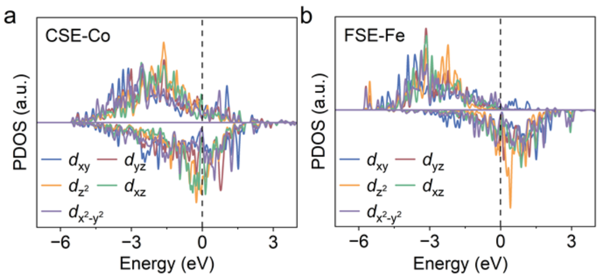

Fig. S2 The PDOS of (a) Co-*d* orbitals in CSE and (b) Fe-*d* orbitals in FSE.

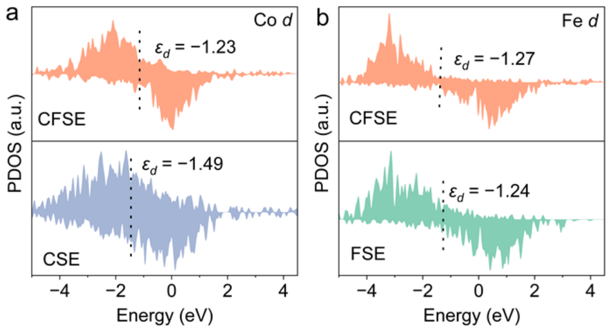

Fig. S3 (a) PDOS of Co in CFSE and CSE. (b) PDOS of Fe in CFSE and FSE.

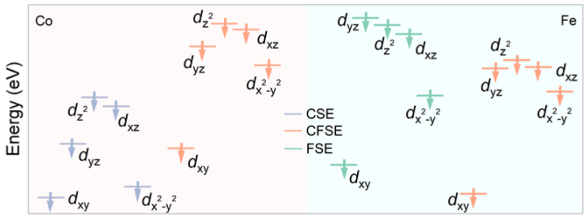

Fig. S4 Schematic diagram of the energy levels of Co-*d* and Fe-*d* orbitals.

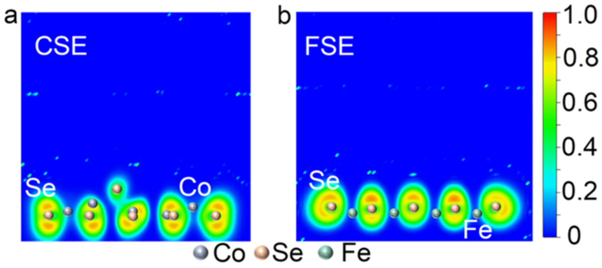

Fig. S5 The ELF of (a) CSE and (b) FSE.

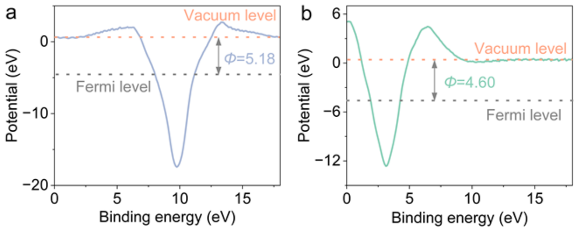

Fig. S6 The work function of (a) CSE and (b) FSE.

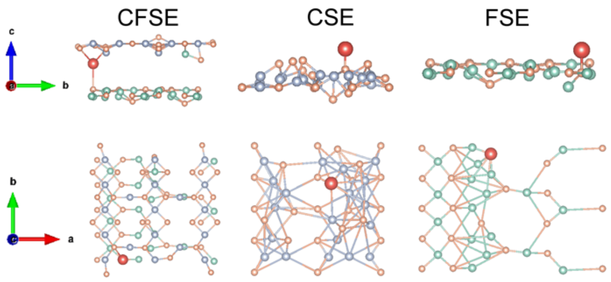

Fig. S7 Adsorption energy models of CFSE, CSE, and FSE.

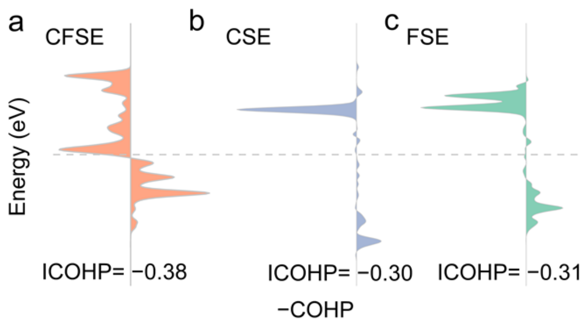

Fig. S8 COHP plots of Na−Se bond and corresponding ICOHP values in (a) CFSE, (b) CSE, and (c) FSE.

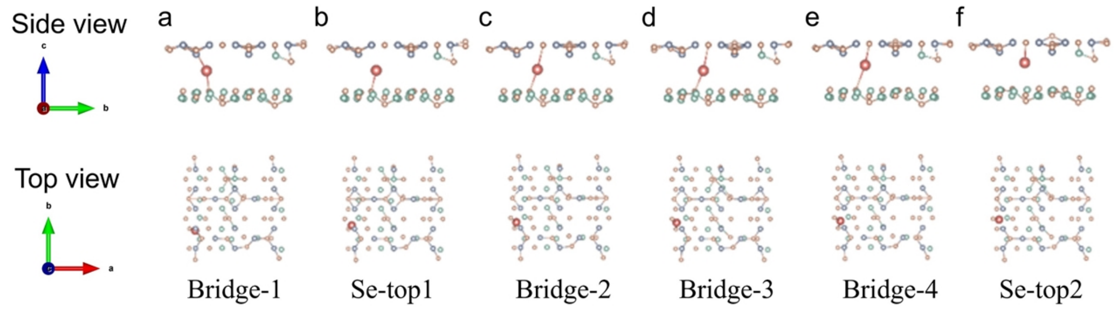

Fig. S9 The diffusion path of sodium ions at the interface of CFSE.

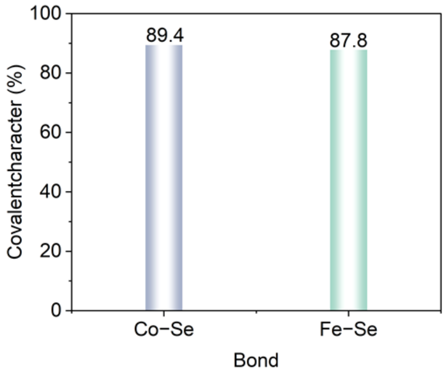

Fig. S10 The covalent characters of Co−Se and Fe−Se bonds.

The covalent character can be calculated by the following equation $(S1)$:

$$\text{Covalentcharacter}\ (\%) = 100 \times \exp\left[-0.25\ (X_a - X_b)^2\right]$$

where X_a and X_b denote the corresponding electronegativity value of the anion and cation.

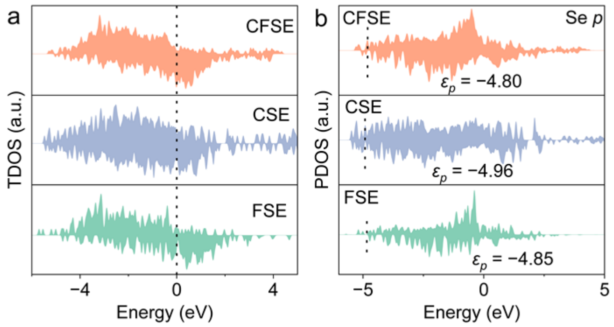

Fig. S11 (a) TDOS of CFSE, CSE, and FSE. (b) PDOS of Se in CFSE, CSE, and FSE.

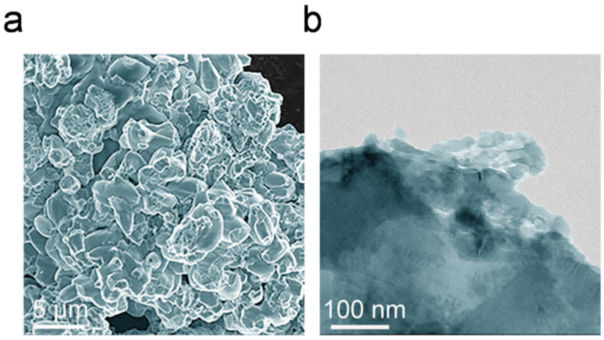

Fig. S12 (a) SEM images and (b) TEM images of CFSE.

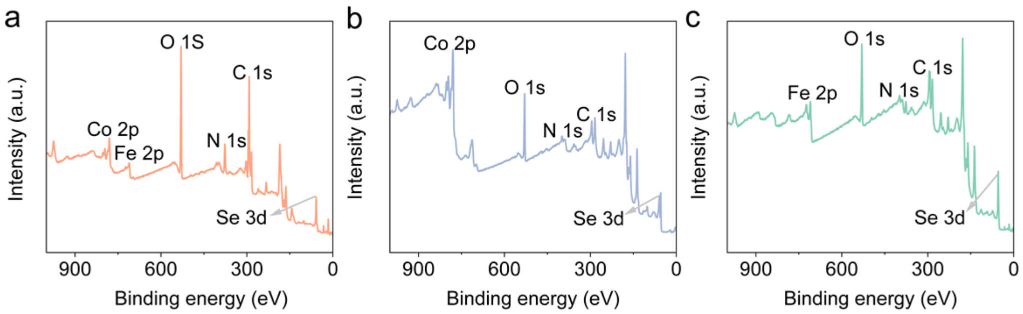

Fig. S13 XPS full spectra of (a) CFSE, (b) CSE, and (c) FSE.

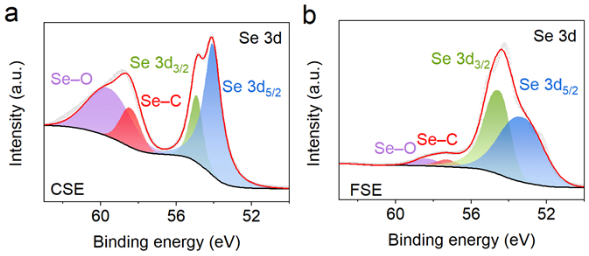

Fig. S14 XPS spectra of Se 3d for (a) CSE and (b) FSE.

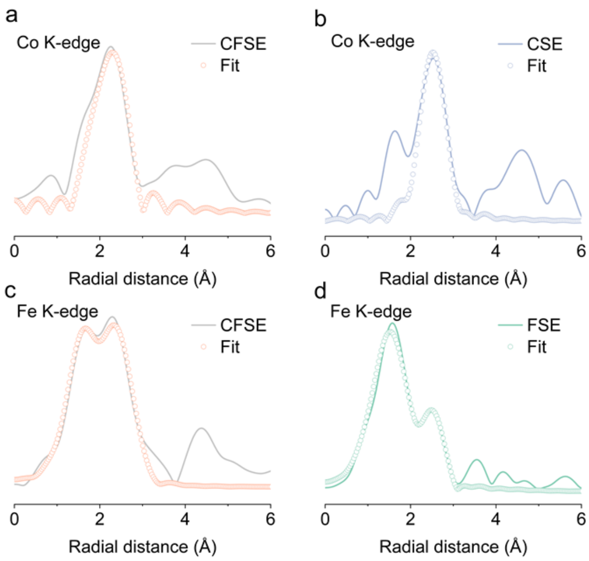

Fig. S15 EXAFS fitting profiles for Co K-edge of (a) CFSE, (b) CSE, and Fe K-edge of (c) CFSE, (d) FSE.

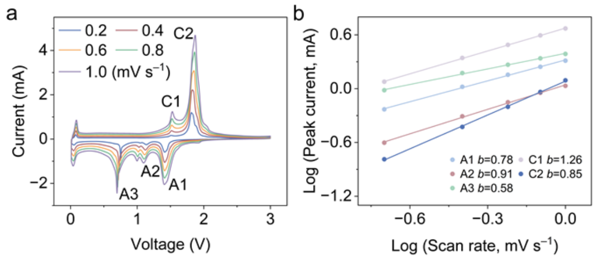

Fig. S16 (a) The CV curves of CSE at different scan rates. (b) *b* values.

The correlation between the peak currents and different scan rates is described by the equation $(S2)$:

$$i = a \cdot v^b \qquad (S2)$$

where *i*, *a* (*b*), and *v* represent the current, constants, and scan rate, respectively.

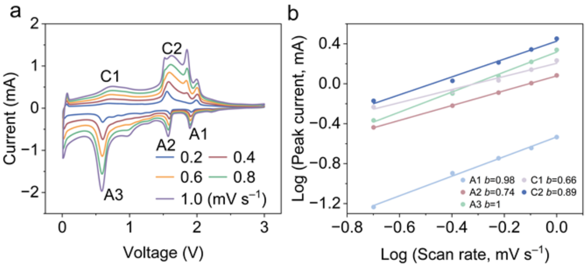

Fig. S17 (a) The CV curves of FSE at different scan rates. (b) *b* values.

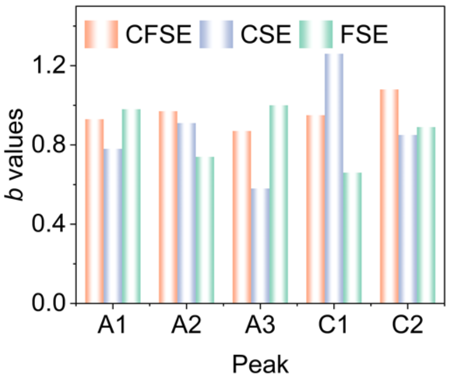

Fig. S18 The *b* values for the CFSE, CSE, and FSE.

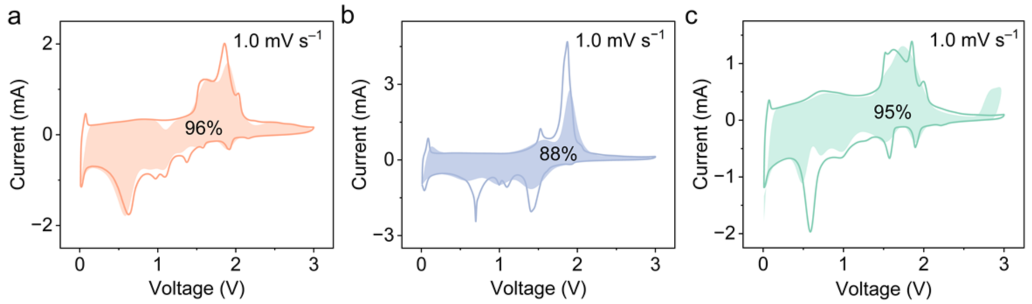

Fig. S19 The proportion of capacitive contribution in the CV curves at 1.0 mV s^{-1} for (a) CFSE, (b) CSE, and (c) FSE.

The capacitive contribution is determined by the equation $(S3)$:

$$i(V) = k_1 v + k_2 v^{\frac{1}{2}} \tag{S3}$$

where i(V), k_1v, and $k_2v^{1/2}$ denote the current at a corresponding potential, capacity contribution of capacitance, and capacity contribution of diffusion.

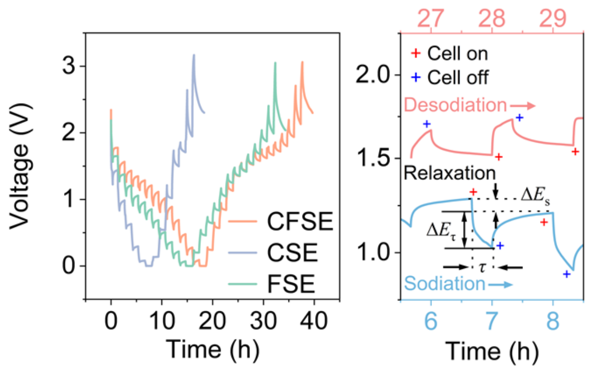

Fig. S20 GITT tests of time–voltage profiles and partial zoom-in profiles for CFSE, CSE, and FSE.

The D_{Na^+} values can be calculated by the following equation $(S4)$:

$$D = \frac{4}{\pi\tau}\left(\frac{m_B V_M}{M_B S}\right)^2\left(\frac{\Delta E_s}{\Delta E_\tau}\right)^2 \qquad (S4)$$

where τ represents the relaxation time; V_M, m_B, and M_B are the molar volume, mass loading, and molar weight of the active materials. S represents the contact area between electrolyte and active materials. ΔE_s and ΔE_τ denote the variation of potential in two consecutive relaxation periods and the variation of potential during a single titration, respectively.

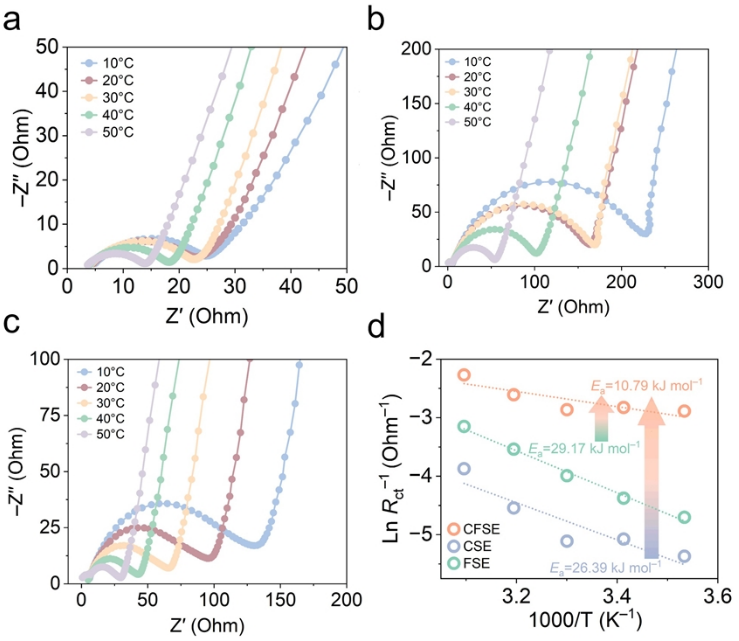

Fig. S21 Nyquist plots of (a) CFSE, (b) CSE, and (c) FSE at various temperatures and (d) E_a values.

The activation energy can be further calculated by the equation $(S5)$:

$$\frac{1}{R_{ct}} = A_0 exp\frac{-E_a}{RT} \qquad (S5)$$

where A_0, E_a, T, and R denote pre-exponential factor, activation energy, absolute temperature, and gas constant, respectively.

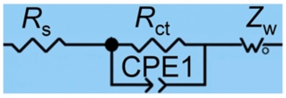

Fig. S22 Equivalent circuit.

Where the R_s represents the cell resistance within the electrolyte, R_{ct} denotes the charge transport resistance. The CPE1 corresponds to constant phase elements, which are associated capacitive responses of electrons at various interfaces, and the Z_w represents Warburg impedance, which is related to Na^+ migration within anode material.

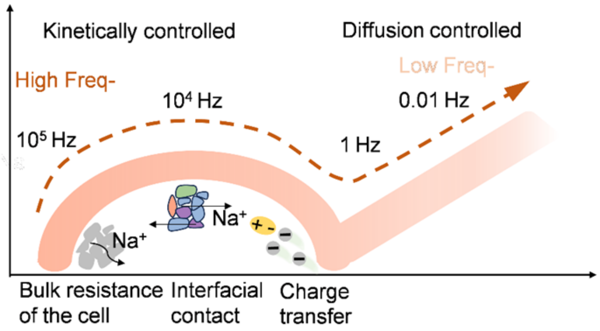

Fig. S23 Schematic diagram of electrochemical behavior across various frequency ranges.

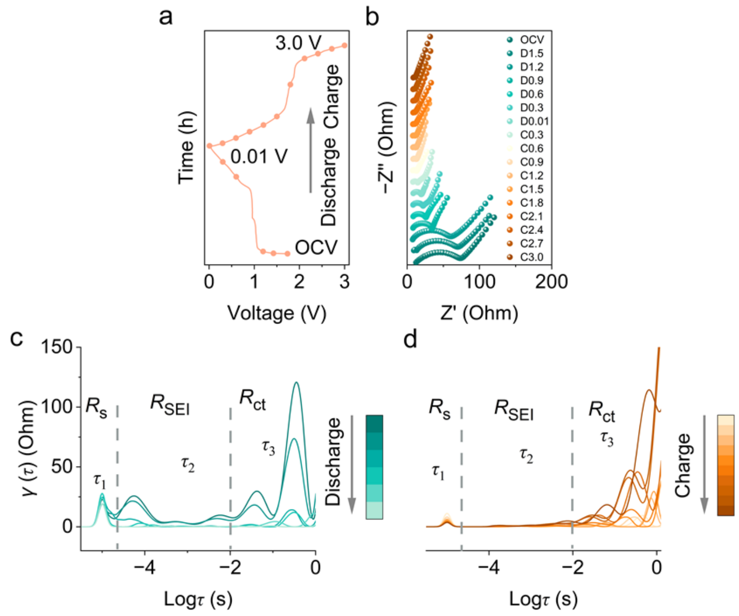

Fig. S24 (a) GCD profile of CFSE (b) In-situ EIS plots of CFSE. DRT curves for the (c) discharge and (d) charge of CFSE.

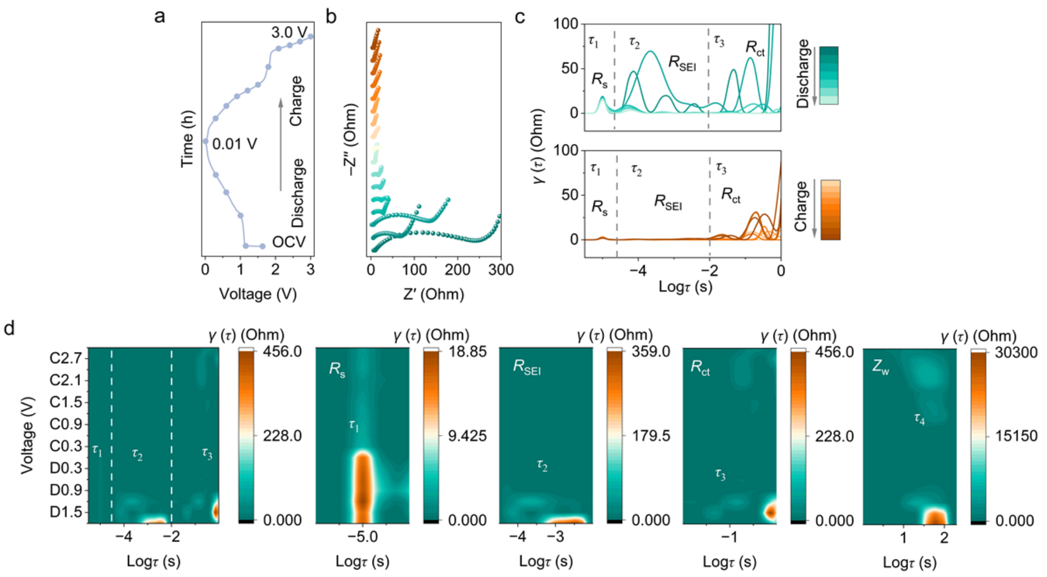

Fig. S25 (a) GCD profile of CSE. (b) In-situ EIS plots of CSE. (c) DRT curves and (d) 2D DRT intensity mapping of CSE.

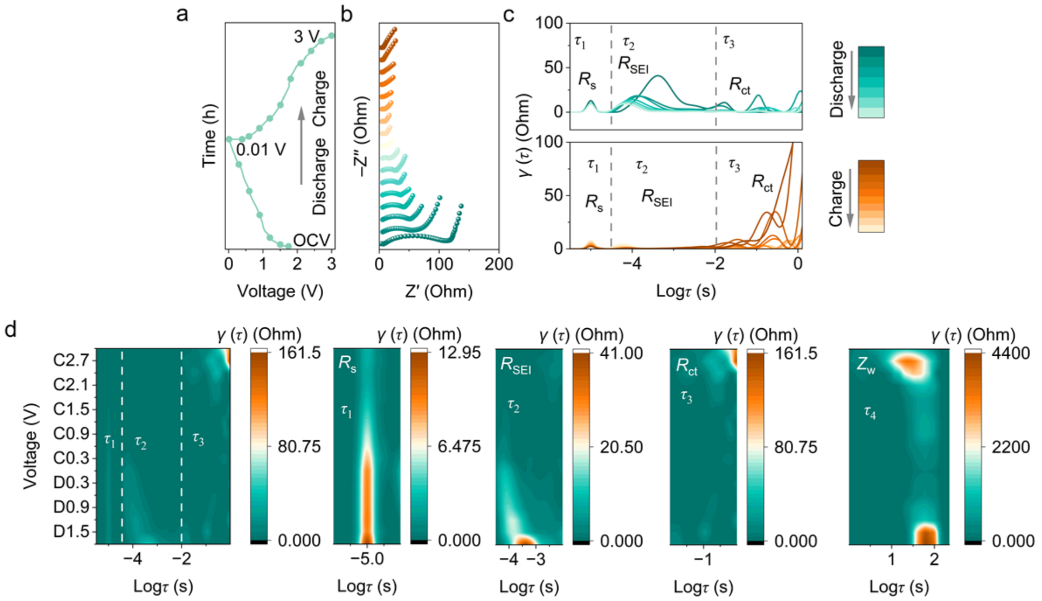

Fig. S26 (a) GCD profile of FSE. (b) In-situ EIS plots of FSE. (c) DRT curves and (d) 2D DRT intensity mapping of FSE.

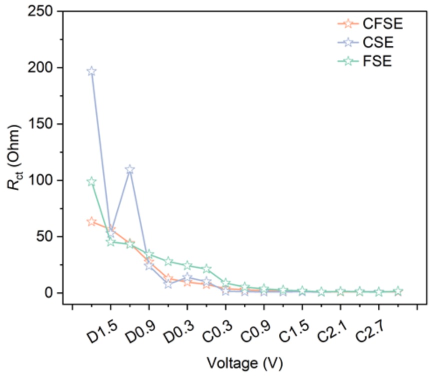

Fig. S27 The fitted impedance values of R_{ct} at various voltages during the first discharge/charge processes.

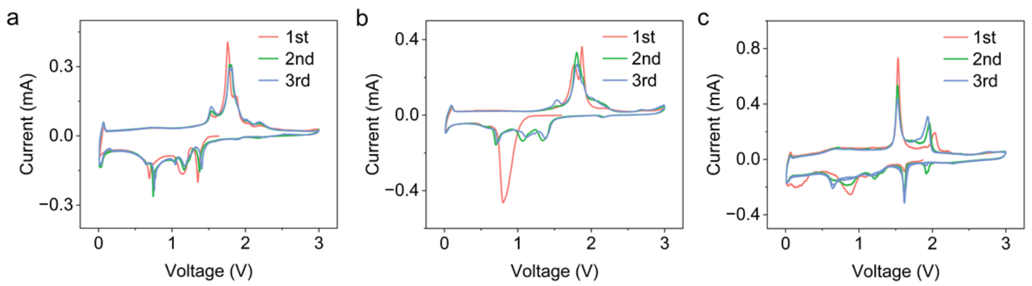

Fig. S28 CV curves of (a) CFSE, (b) CSE, and (c) FSE at 0.1 mV s^{-1}.

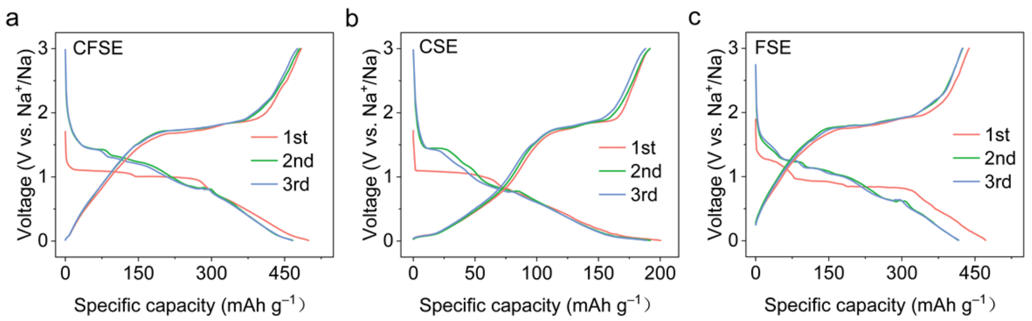

Fig. S29 The GCD profiles of (a) CFSE, (b) CSE, and (c) FSE at 0.1 A g^{-1}.

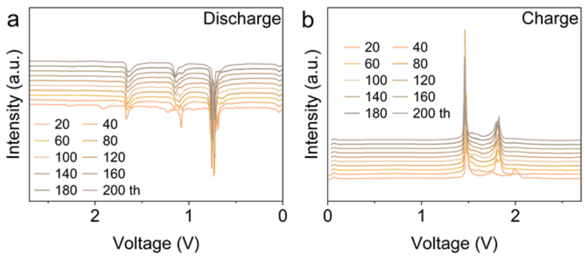

Fig. S30 The dQ/dV curves of CFSE for the first 200 cycles.

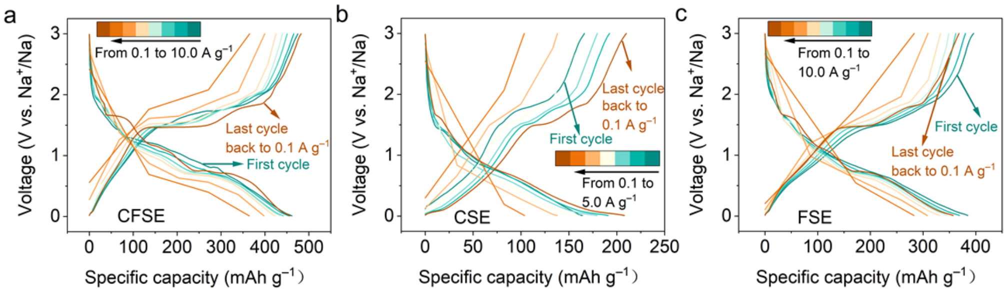

Fig. S31 The GCD curves of the (a) CFSE, (b) CSE, and (c) FSE at various current densities.

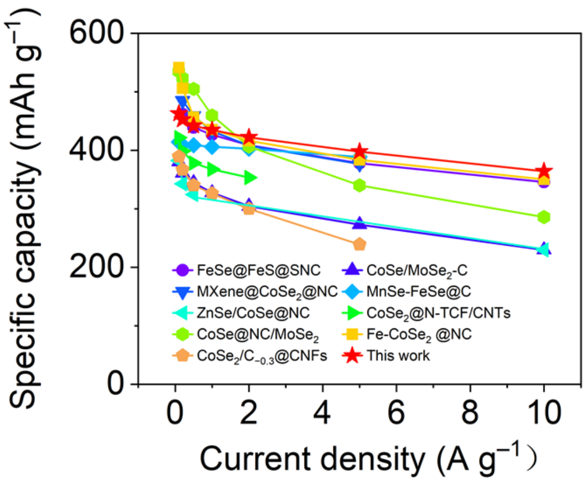

Fig. S32 Comparisons of rate performances between CFSE and the reported bimetallic selenide anodes.

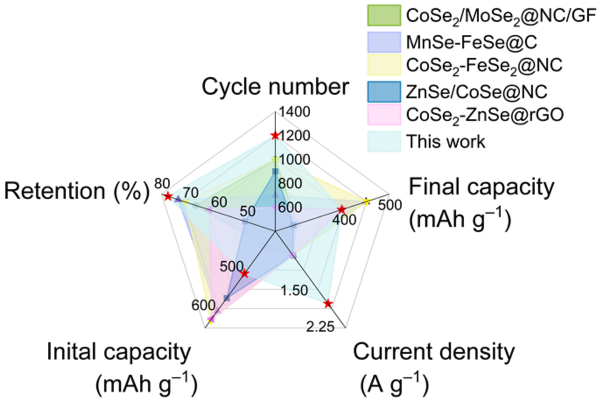

Fig. S33 Comparison of long-term cycling performance of CFSE with other conversion anode materials for SIBs.

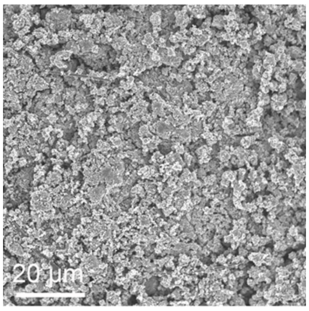

Fig. S34 SEM image of CFSE after cycling.

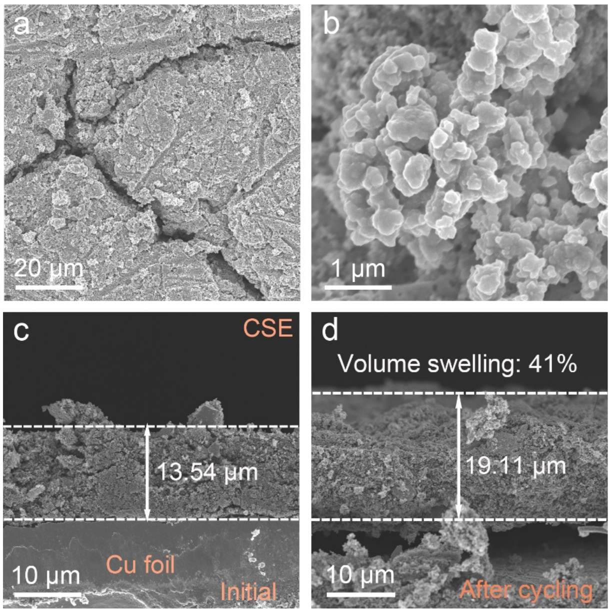

Fig. S35 (a, b) SEM image of CSE after cycling. Cross-sectional SEM images of CSE (c) before and (d) after cycling.

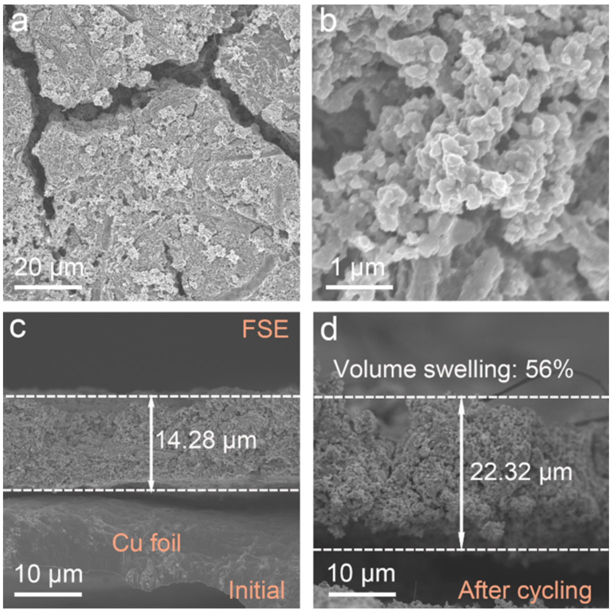

Fig. S36 (a, b) SEM image of FSE after cycling. Cross-sectional SEM images of FSE (c) before and (d) after cycling.

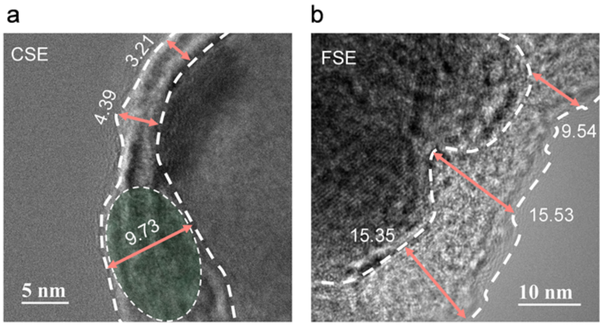

Fig. S37 HRTEM images of (a) CSE, and (b) FSE electrodes at 0.1 A g^{-1}.

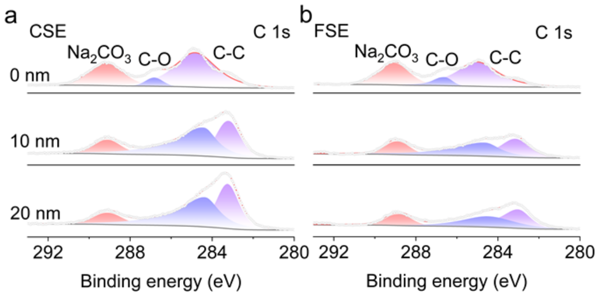

Fig. S38 XPS spectra of C 1s after the 10th cycles in the (a) CSE and (b) FSE.

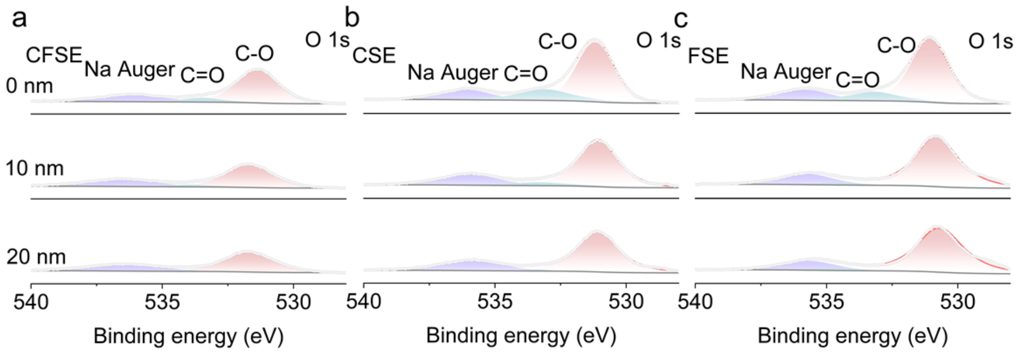

Fig. S39 XPS spectra of O 1s after the 10th cycles in the (a) CFSE, (b) CSE, and (c) FSE.

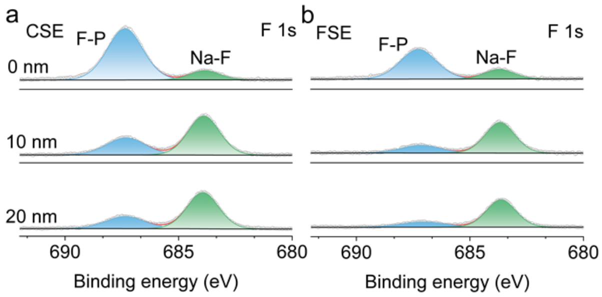

Fig. S40 XPS spectra of F 1s after the 10th cycles in the (a) CSE and (b) FSE.

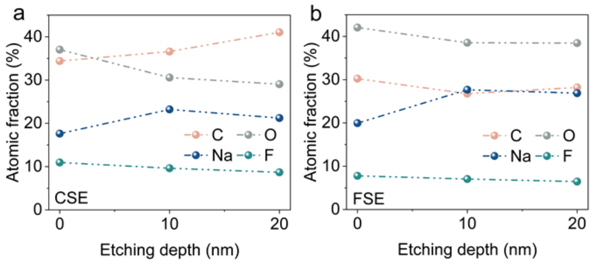

Fig. S41 Atomic fraction of C, O, F, and Na after different etching depths in the (a) CSE and (b) FSE.

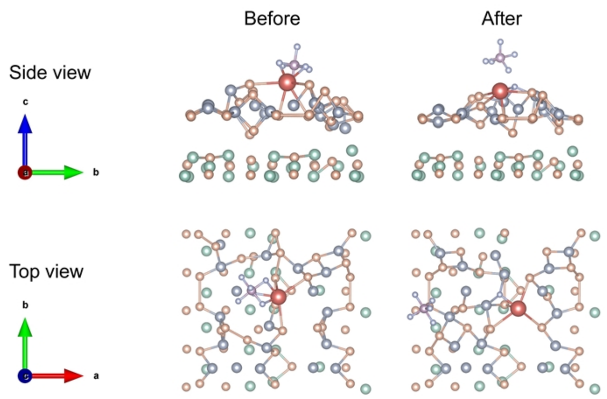

Fig. S42 The optimized models of $NaFP_6$ decomposition on the surface of CFSE.

i. $CH_3OCH_2CH_2OCH_3 + 2e^- + 2Na^+ \rightarrow 2CH_3ONa + CH_2{=}CH_2\uparrow$

ii. $H_2O + e^- + Na^+ \rightarrow NaOH + 0.5H_2\uparrow$ $NaOH + e^- + Na^+ \rightarrow Na_2O + 0.5H_2\uparrow$

Fig. S43 Reaction equations of gas generation during the discharge process.

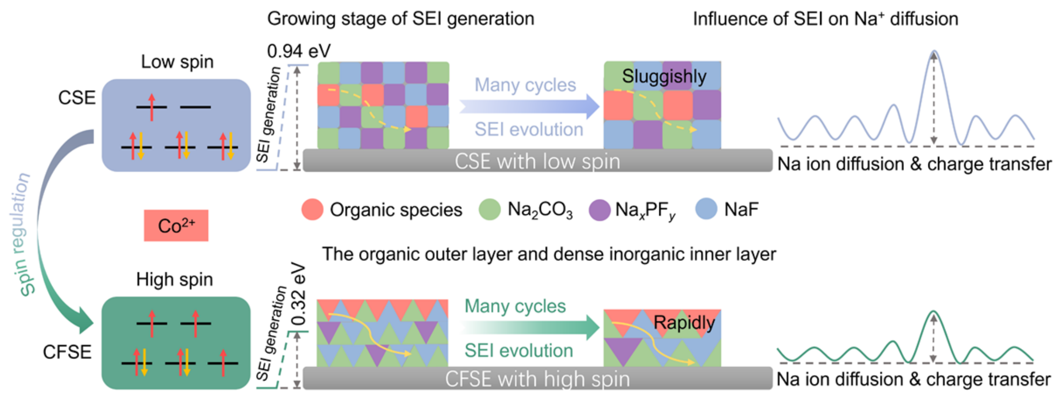

Fig. S44 Schematic diagram of formation and evolution of SEI structures and potential diffusion pathway of Na ion in CSE with low spin and CFSE with high spin electrodes, respectively.

Table S1. Curve fit parameters of EXAFS for various samples.

Sample	Path	*CN*[a]	*R* (Å)[b]	σ^2 (Å^2)[c]	ΔE_0 (eV)[d]	*R* factor[e]
CSE	Co-Se	3.17±0.99	2.99±0.02	0.015	−10.445	0.017
CFSE	Co-Se	2.99±1.50	2.52±0.03	0.021	12.145	0.016
FSE	Fe-Se	2.08±0.48	2.13±0.02	0.055	−25.485	0.009
	Fe-Se	3.09±1.64	2.99±0.02	0.035	−25.485	
CFSE	Fe-Se	2.90±1.48	2.21±0.02	0.035	−5.490	0.009
	Fe- Fe	3.44±1.43	2.95±0.04	0.036	−5.490	

CN[a] is the coordination number; *R* (Å)[b] is the distance to the neighboring atoms; σ^2 (Å^2)[c] is the Debye-Waller factor; ΔE_0 (eV)[d] is the inner potential correction; *R* factor is the residual factor. S_0^2 was fixed to 1.

Table S2. The rate performance comparisons of CFSE with previously reported selenium-based heterojunction anode materials for SIBs.

Current density (A g^{-1}) / Capacity (mAh g^{-1}) / Material	0.1	0.2	0.5	1	2	5	10	Ref.
FeSe@FeS@SNC	/	462	439	427	409	378	346	[5]
CoSe/$MoSe_2$-C	380.3	360.9	344.8	327.7	304.5	273.1	230.0	[6]
MXene@$CoSe_2$@NC	/	485	459	433	408	377	/	[7]
MnSe-FeSe@C	414.3	409.5	408.7	406.1	402.9	388.8	/	[8]
ZnSe/CoSe@NC	382.5	342.7	324.7	/	/	/	229.8	[9]
$CoSe_2$@N-TCF/CNTs	421.7	399.0	378.2	367.7	353.7	/	/	[10]
CoSe@NC/$MoSe_2$	534.7	523.8	504.9	459.8	406.6	340.2	286.1	[11]
Fe-$CoSe_2$@NC	541.5	506.0	456.8	435.6	416.6	383.7	350.3	[12]
$CoSe_2$/$C_{-0.3}$@CNFs	389.4	367.0	340.2	326.1	300.0	239.2	/	[13]
CFSE	461.7	454.2	442.0	434.8	422.3	398.0	364.4	This work

Table S3. The comparison of cycling of CFSE with previously reported selenium-based heterojunction anode materials for SIBs.

Materials	Cycle number	Current density (A g^{-1})	Initial capacity (mAh g^{-1})	Final capacity (mAh g^{-1})	Capacity retention (%)	Ref.
$CoSe_2/MoSe_2$@NC/GF	1000	1	438	298	68	[14]
MnSe-FeSe@C	700	1	606.0	449.2	74.1	[8]
$CoSe_2$-$FeSe_2$@NC	1000	1	629.3	450.6	71.6	[15]
ZnSe/CoSe @NC	900	1	572.7	289.7	50.6	[9]
$CoSe_2$-ZnSe@rGO	600	1	626.3	395.2	63.1	[16]
CFSE	1200	2	419.4	395.8	94.4	This work

Reference

1 G. Kresse, J. Furthmüller, *Comput. Mater. Sci.*, 1996, **6**, 15-50.

2 P.E. Blöchl, *Phys. Rev. B*, 1994, **50**, 17953-17979.

3 J.P. Perdew, K. Burke, M. Ernzerhof, *Rev. Lett.*, 1996, **77**, 3865-3868.

4 S. Grimme, J. Antony, S. Ehrlich, H. Krieg, *J. Chem. Phys.*, 2010, **132**, 154104.

5 Z.-H. Sun, D.-Y. Qu, D.-X. Han, Z.-Y. Gu, J.-Z. Guo, X.-X. Zhao, Y.-M. Ma, B.-L. Zhao, Z.-Q. Song, X.-L. Wu, L. Niu, *Adv. Mater.*, 2024, **36**, 2308987.

6 J. Li, Y. He, Y. Dai, H. Zhang, Y. Zhang, S. Gu, X. Wang, T. Gao, G. Zhou, L. Xu, *Adv. Funct. Mater.*, 2024, **34**, 2406915.

7 R. An, H. Niu, C. Wang, Z. Liu, B. Gao, *J. Energy Storage*, 2024, **91**, 111968.

8 T. Liu, L. Xu, X. Wang, H. Lv, B. Zhu, J. Yu, L. Zhang, *J. Colloid Interface Sci.*, 2024, **672**, 43-52.

9 L. Gao, M. Cao, C. Zhang, J. Li, X. Zhu, X. Guo, Z. Toktarbay, *Adv. Compos. Hybrid Mater.*, 2024, **7**, 144.

10 L. Wang, M. Hu, C. Lin, J. Feng, W. Yan, *J. Energy Storage*, 2023, **67**, 107584.

11 J. Feng, S.-h. Luo, P.-w. Li, Y.-c. Lin, L. Zhang, Q. Wang, Y.-h. Zhang, *Appl. Surf. Sci.*, 2023, **619**, 156775.

12 Z. Huang, J. Liu, S. Wang, F.-L. Li, J. Yang, H. Gu, *J. Alloys Compd.*, 2024, **997**, 174987.

13 X. Liu, D. Xue, R. Ren, T. Ou, T. Sun, Y. Han, F. Jin, Y. Li, Y. Zhao, J. Zhang, *Chem. Eng. J.*, 2024, **496**, 153790.

14 Y. Li, W. Li, M. Zhang, Y. Zhuang, H. Li, Z. Pan, H. Min, T.-Y. Chen, H.-Y. Chen,

H. Yang, J. Wang, *Small*, 2024, **20**, 2405819.

15 H. Tian, Y. Li, Z. Sun, X. Fu, L. Chen, Y. Chen, D. Sun, B. Zhou, H. Yang, *J. Colloid Interface Sci.*, 2025, **679**, 40-49.

16 H. Tian, Z. Sun, L. Ren, Y. Jin, D. Wang, Y. Wei, H. Chen, K. Liu, Y. Chen, H. Yang, *J. Colloid Interface Sci.*, 2024, **658**, 827-835.
